# Cortico-cortical feedback engages active dendrites in visual cortex

**DOI:** 10.1038/s41586-023-06007-6

**Published:** 2023-05-03

**Authors:** Mehmet Fişek, Dustin Herrmann, Alexander Egea-Weiss, Matilda Cloves, Lisa Bauer, Tai-Ying Lee, Lloyd E. Russell, Michael Häusser

**Affiliations:** Wolfson Institute for Biomedical Research and Department of Neuroscience, Physiology and Pharmacology, University College London, London, UK

**Keywords:** Neuroscience, Sensory processing

## Abstract

Sensory processing in the neocortex requires both feedforward and feedback information flow between cortical areas^1^. In feedback processing, higher-level representations provide contextual information to lower levels, and facilitate perceptual functions such as contour integration and figure–ground segmentation^2,3^. However, we have limited understanding of the circuit and cellular mechanisms that mediate feedback influence. Here we use long-range all-optical connectivity mapping in mice to show that feedback influence from the lateromedial higher visual area (LM) to the primary visual cortex (V1) is spatially organized. When the source and target of feedback represent the same area of visual space, feedback is relatively suppressive. By contrast, when the source is offset from the target in visual space, feedback is relatively facilitating. Two-photon calcium imaging data show that this facilitating feedback is nonlinearly integrated in the apical tuft dendrites of V1 pyramidal neurons: retinotopically offset (surround) visual stimuli drive local dendritic calcium signals indicative of regenerative events, and two-photon optogenetic activation of LM neurons projecting to identified feedback-recipient spines in V1 can drive similar branch-specific local calcium signals. Our results show how neocortical feedback connectivity and nonlinear dendritic integration can together form a substrate to support both predictive and cooperative contextual interactions.

## Abstract (web summary)

Feedback influence from a higher visual area to primary visual cortex in mice engages nonlinear dendritic integration.

## Main

To determine how cortico-cortical feedback modifies activity in the recipient circuit, we must solve two problems. First, we must map feedback connectivity and, second, we must understand how feedback is integrated at the single-cell level. A substantial proportion of feedback inputs innervate pyramidal cell apical dendrites in layer 1 (refs. ^1,4^), in which inputs are too distant to effectively influence the soma passively^5^. However, feedback may recruit the active properties of apical dendrites to compensate for this distance-dependent attenuation^5^, providing a layer of nonlinear processing^6,7^. To map feedback connections and determine whether they can drive active dendritic processes, we focused on V1 and one of its prominent feedback sources—LM, which is considered to be the mouse homologue to primate V2 (ref. ^8^). We studied layer 5 intratelencephalic (IT) neurons, which project to other cortical areas^9^, possess apical dendrites capable of intrinsic electrogenesis^10^ and can be targeted using the *Tlx3-cre* transgenic mouse line^9^.

## Circuit organization of feedback

Feedback projections from higher visual areas to any given location in V1 cover a region of visual space that is much larger than the size of individual receptive fields^11,12^ and target excitatory as well as inhibitory neurons^13^. Consistent with this, in vivo microstimulation^14^, as well as silencing^15–17^ of feedback, can have both facilitating and suppressive effects. However, it is unclear whether the relative retinotopic locations of the feedback source and target relate to the sign of feedback influence.

**Fig. 1:**
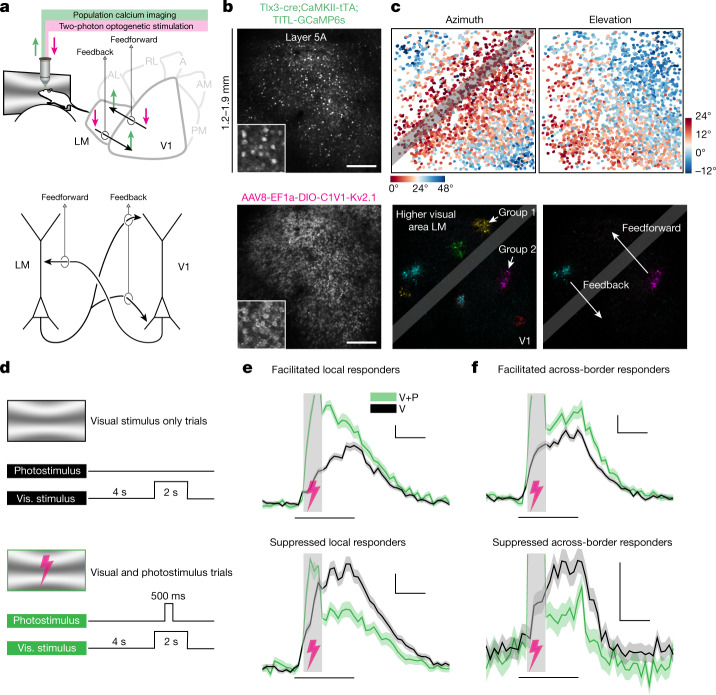
Mesoscale mapping of interareal functional connectivity in mouse visual cortex using simultaneous two-photon optogenetic stimulation and two-photon calcium imaging. **a**, Top, illustration of the experimental set-up to examine interareal functional connectivity between V1 and LM. Bottom, schematic of pyramidal neurons with layer-specific projection preferences. A, anterior higher visual area; AL, anterolateral higher visual area; AM, anteromedial higher visual area; PM, posteromedial higher visual area; RL, rostrolateral higher visual area. **b**, Mean two-photon images for one example FOV, showing the large spatial extent of expression. Top, GCaMP6s expression driven transgenically. Bottom, C1V1 expression, driven using adeno-associated viruses (AAVs). Insets: representative cell bodies. Scale bars, 250 μm. **c**, Top, cellular-resolution retinotopic mapping across V1 and LM with a large FOV, obtained using sparse noise stimulation and two-photon population imaging. The grey bar delineates a 150-μm-wide border zone, which was excluded from stimulation and responder detection. Bottom, the mean photostimulation response of example stimulation groups (pixelwise stimulus-triggered averages). **d**, Trial structure. Full field sinusoidal gratings were presented either alone, or paired with a photostimulus. Responses to the two trial types were compared to detect responders. Vis., visual. **e**, Example local responders from one session. Data are mean ± s.e.m. across trials. Black lines represent presentation of the visual stimulus. Grey bars with lightning symbols represent the photostimulus. Both examples are from LM. V, visual stimulus only; V+P, visual stimulus and photostimulus. **f**, Example across-border (in the area opposite the stimulated one) responders from one session. Facilitated example from LM, suppressed from V1. For **e** and **f**, scale bars, 1 s (horizontal) and 0.1 Δ*F*/*F* (vertical).

**Fig. 2:**
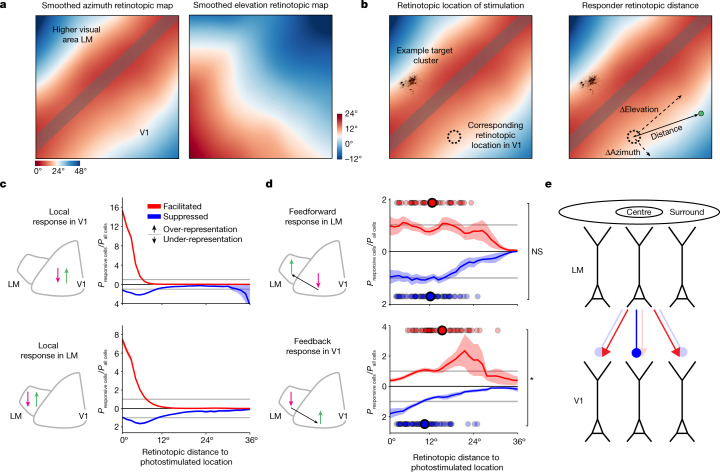
Cortico-cortical feedback is relatively suppressive of topographically matched centre locations and relatively facilitating of mismatched surround locations. **a**, Left, retinotopic map in azimuth used to assign recorded neurons to a cortical area was obtained by smoothing cellular-resolution maps constructed using sparse noise stimuli and two-photon population imaging. Right, smoothed retinotopic map in elevation. **b**, Left, example photostimulation group in LM (probing functional connectivity in the feedback direction) and the corresponding retinotopic location in V1. Right, measurement of the absolute retinotopic (rather than physical) distance of an example across-border responder to a photostimulated cluster. **c**, The probability distribution of responder retinotopic distances divided by the probability distribution of distances for all available neurons. Data are mean ± s.e.m. across stimulation groups. *n* = 129 (LM) and *n* = 180 (V1) clusters from 42 sessions in 11 animals. The horizontal grey lines mark *y* = 1 and represent uniform spatial sampling. Suppressed responders are plotted downwards by convention. Red arrow indicates the location of stimulation, the green arrow indicates the location of responders measured. Top, stimulation and readout in V1. Bottom, stimulation and readout in LM. **d**, The same as in **c**, but for interareal stimulation and readout, and also including the average retinotopic location of responders for each stimulation group (red and blue dots), which were used to make the comparison. Top, stimulation in V1 and readout in LM (feedforward). Bottom, stimulation in LM and readout in V1 (feedback). Feedforward-facilitated and feedforward-suppressed responders do not differ in spatial distribution (centroids over stimulation groups, rank-sum test, *P* = 0.61). Feedback-facilitated and feedback-suppressed responders are displaced relative to each other (rank-sum test, *P* = 9.2 × 10^−5^). NS, not significant. **e**, Schematic illustrating the suppressive feedback from retinotopically aligned and facilitating feedback from retinotopically offset projections.

**Extended Data Fig. 1:**
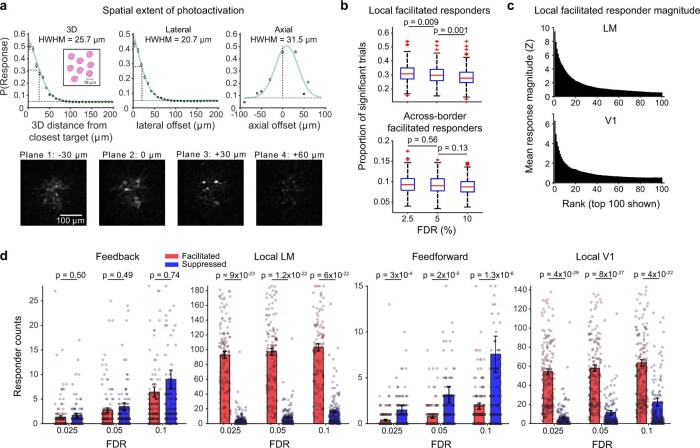
Photoactivation parameters, response magnitude and reliability. **a**, Spatial extent of two-photon optogenetic photoactivation. Top, response probability by distance from targeted locations. Curves obtained from the experiments that produced data in Figs. 1 & 2. Bottom, pixelwise photostimulus-triggered response averages of one example photostimulation group in three dimensions. **b**, Response reliability of facilitated responders in the stimulated area (top, “local”) and in the other area (bottom, “across”) as a function of false discovery rate (n = 310 stimulation groups, two-sided Wilcoxon rank sum test). Boxes indicate median, 75^th^ and 25^th^ percentile, whiskers indicate upper and lower adjacent values. **c**, Most locally facilitated responders are weakly activated, with a small number being strongly activated. **d**, The number of facilitated and suppressed responders as a function of false discovery rate. Bar plots represent mean ± s.e.m, dots indicate individual stimulation groups (n = 130 in LM and 180 in V1). Statistical test used was a Wilcoxon signed rank test between facilitated and suppressed responder counts. Note that the y-axis was set to the 95^th^ percentile of the data for FDR 0.1 to facilitate visualization.

To map long-range functional connectivity across areas at cellular resolution, we used simultaneous two-photon optogenetics and calcium imaging at the meso-scale (Fig. 1a (top)), extending approaches that focused on local connectivity^18^. We co-expressed the calcium indicator GCaMP6s and the soma-targeted excitatory opsin C1V1(t/t)-Kv2.1 in layer 5 IT neurons across V1 and LM (Fig. 1b). We first mapped visual receptive fields and generated retinotopic maps that delineated the border between the two areas (Figs. 1c and 2a). We then holographically photostimulated clustered groups of neurons (6–14 targets) and simultaneously recorded population activity at cellular resolution across both cortical areas using two-photon calcium imaging (Fig. 1c and Extended Data Fig. 1a–c). We performed photostimulation simultaneously with visual stimulation, enabling us to resolve small changes in physiological patterns of activity due to our manipulation.

**Extended Data Fig. 2:**
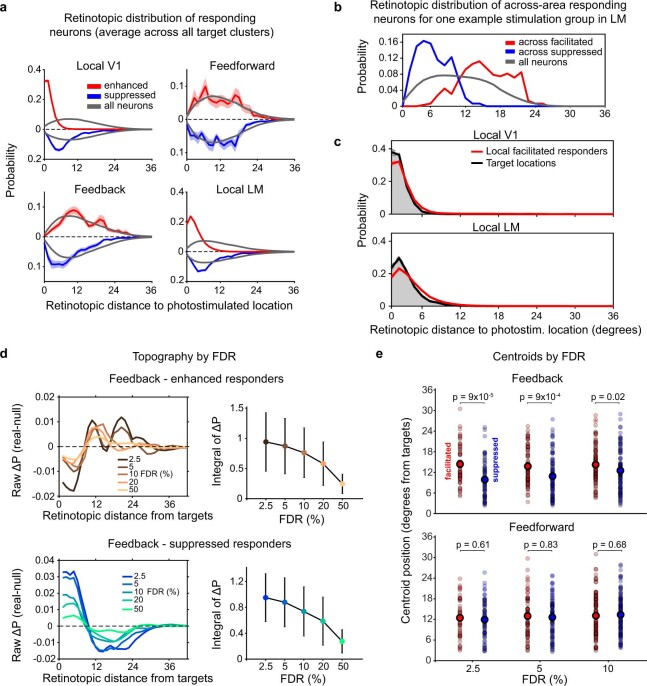
Topographic map weighting and dependence on FDR. **a**, Un-weighted retinotopic distance distributions of responders. These distributions are averages across all experiments. Data shown in Fig. 2c,d are weighted, where the responder retinotopic distribution obtained for each stimulation group is weighted by the corresponding “all neurons” distribution for that stimulation group. **b**, Un-weighted distance distributions of responders (as in a) for one example stimulation group (across-area facilitated and suppressed followers after LM photostimulation). **c**, Un-weighted distance distributions of locally facilitated responders and target locations. Dashed lines s.e.m. across stimulation groups. **d**, Topographic biases do not depend on FDR. Top left, the difference between un-weighted retinotopic distance distributions for facilitated feedback responders and their corresponding “all neurons” distributions. Bias across distance changes gradually as a function of FDR. Right, the integral of the difference plotted on the left as a function of FDR (mean ± s.d., n = 129 stimulation groups). **e**, Each dot is the centroid of the retinotopic locations of responders recruited by one stimulation group (only stimulation groups with ≥ 1 across border responder, Feedback: n = 51/74/103 groups (FDR 2.5%, 5%, 10%) facilitated, n = 64/90/106 suppressed; Feedforward: n = 42/63/112 facilitated, n = 61/83/116 suppressed). Responder distributions are displaced between facilitated and suppressed responders in the feedback direction but not in the feedforward direction (see also Fig. 2; Wilcoxon rank sum test). This comparison remains significant across different FDR thresholds for feedback.

To identify photostimulus-responsive neurons (responders), we compared each neuron’s responses to visual stimulation with and without photostimulation (Fig. 1d) by performing a Wilcoxon rank-sum test and controlling for the false-discovery rate (FDR) across all neurons (Methods and Extended Data Figs. 1d and 2a–e). This procedure yielded both facilitated and suppressed neurons in both the directly targeted ‘local’ and the other ‘across-border’ area (Fig. 1e,f; FDR = 2.5%, mean ± s.d. number of neurons across stimulation groups locally in V1, 54.2 ± 41.2 (facilitated), 6.1 ± 17.4 (suppressed); local LM, 93.7 ± 52.9 (facilitated), 5.6 ± 10.7 (suppressed); V1 to LM, 0.4 ± 1.2 (facilitated), 1.5 ± 6.3 (suppressed); LM to V1, 1.3 ± 3.3 (facilitated), 1.7 ± 4.8 (suppressed)). To reveal the retinotopic distribution of these responders, we computed their retinotopic distance from the photostimulated location, represented by all locally facilitated responders (source neurons; Methods and Fig. 2b). For visualization, we weighted the resulting distribution of retinotopic distances by the distribution of all neurons in the field of view (FOV) and averaged across photostimulation groups. This illustrates the over- or under-representation of feedback-facilitated and feedback-suppressed neurons across retinotopic distance from the photostimulation site (Fig. 2c,d).

**Extended Data Fig. 3:**
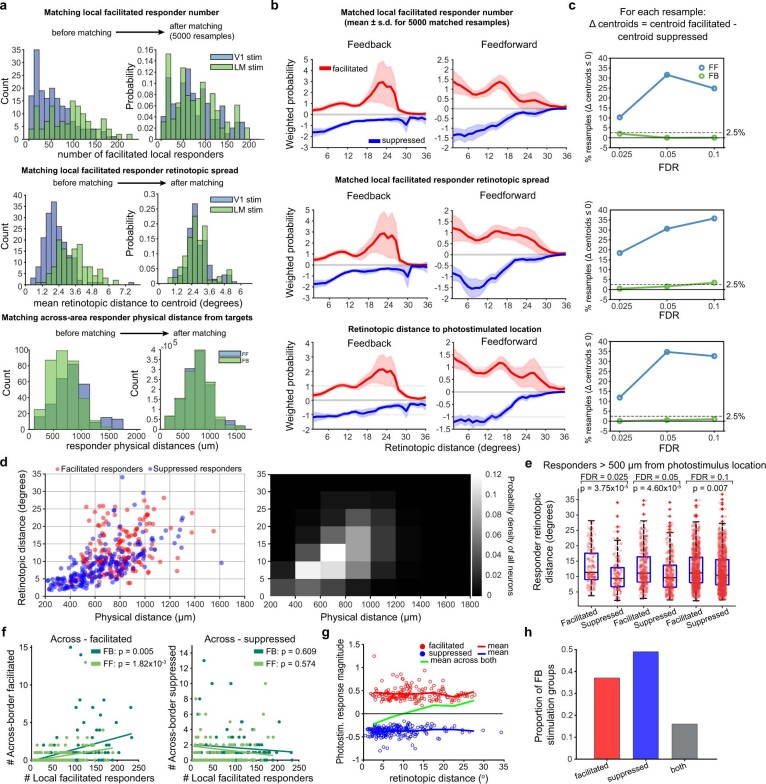
Differences in stimulation do not account for across-border response topography. **a**, Procedure for matching local facilitated responder numbers (top), retinotopic spread (middle) and across-area responder physical distances from target location (bottom). Left, distributions for all stimulation groups before matching, which was performed by randomly sampling the overlapping segment of the distributions. Right, distributions after matching (see Methods). **b**, Across-border response topography in the feedback (left) and feedforward (right) direction computed from resamples (mean ± s.d. across samples). Relative patterns of facilitation and suppression are maintained (Fig. 2). **c**, Proportion of resamples with a negative difference between centroids of weighted probability distributions for facilitated and suppressed neurons as a function of FDR. Negative difference indicates suppressed neurons displaced relative to facilitated. **d**, Left, retinotopic distance of responder to target location as a function of physical distance of responder to target location. Right, density plot showing the availability of all neurons. **e**, Retinotopic distance distributions of facilitated and suppressed responders more than 500 μm away from the target location. **f**, Number of across-border facilitated (left) and suppressed (right) neurons as a function of the number of local responders. **g**, Photostimulation response magnitude as a function of retinotopic distance for all responders at FDR = 2.5%. Response magnitude is the difference between mean responses to the V+P trials and V trials. There is no effect of distance on response magnitude (ANOVA, p = 0.34 facilitated, p = 0.35 suppressed). Solid lines indicate binned and averaged response magnitude. **h**, Probability of a feedback stimulation group producing at least one suppressed, at least one facilitated and at least one of each type of responder. Probability of finding both is not significantly less than would be expected based on the individual rates (permutation test, p = 0.22).

Using this approach, we examined how local, feedforward (V1 to LM) and feedback (LM to V1) functional connectivity depends on relative topography. Locally, in both V1 and LM, photostimulation caused a spatially restricted facilitation consisting of directly targeted and synaptically recruited neurons (Extended Data Fig. 2). In addition, photostimulation of V1 or LM recruited a local surround of suppression, consistent with recent reports^18^ of local functional connectivity in V1 (Fig. 2c). In the feedforward direction, facilitated and suppressed responders were distributed similarly (Fig. 2d (top) and Extended Data Fig. 2e). Responders in the feedback direction exhibited a topographic organization: the unweighted locations of facilitated and suppressed responders were significantly displaced relative to each other, with suppressed responders retinotopically closer and facilitated responders farther away from the photostimulated retinotopic location (Fig. 2d (bottom) and Extended Data Fig. 2d,e). However, these biases were not absolute: facilitated and suppressed responders overlapped across a wide range of retinotopic distances, in which feedback had both a positive and negative influence (Fig. 2d). These results did not depend on the FDR (Extended Data Fig. 2d,e), physical distance, or differences in the stimulation strength between V1 and LM (Extended Data Fig. 3a–e). The number of responders did depend on the stimulation strength but the magnitude of the response did not vary retinotopically (Extended Data Fig. 3f,g). Finally, individual feedback stimulation groups could generate both facilitated and suppressed across-area responders (Extended Data Fig. 3h). Overall, these results show that the functional influence of feedback from LM to V1 is retinotopically organized, that is, it depends on the retinotopic alignment between source and target. When connections from LM to V1 are between regions that are responsive to the same portion of visual space (retinotopic distance = 0°), LM influence over V1 is relatively more suppressive. When connections are between regions that are responsive to stimuli offset from each other in visual space (retinotopic distance ≫ 0°), LM influence over V1 is relatively more facilitating. In other words, feedback influence has a relatively suppressive centre and a relatively facilitating surround (Fig. 2e).

## Surround stimuli evoke dendritic events

How feedback exerts its influence depends not only on connectivity, but also on synaptic integration by the recipient neurons. Although feedback can act through both basal and apical dendritic arbours, integration in apical dendrites is particularly complex—their remoteness from the soma impedes passive integration, but enables them to operate as partially independent compartments that can produce regenerative events associated with calcium entry^7,19,20^. These events could amplify feedback influence^5,19,21,22^, as well as perform thresholding and gain control operations^23,24^. It is therefore critical to determine the conditions under which such events are recruited in visual cortex in vivo^25^. On the basis of the feedback connectivity that we observed, we predicted that apical tufts in V1 should receive relatively more facilitating feedback from their retinotopic surround, potentially recruiting regenerative dendritic events to promote their influence on neuronal output.

**Fig. 3:**
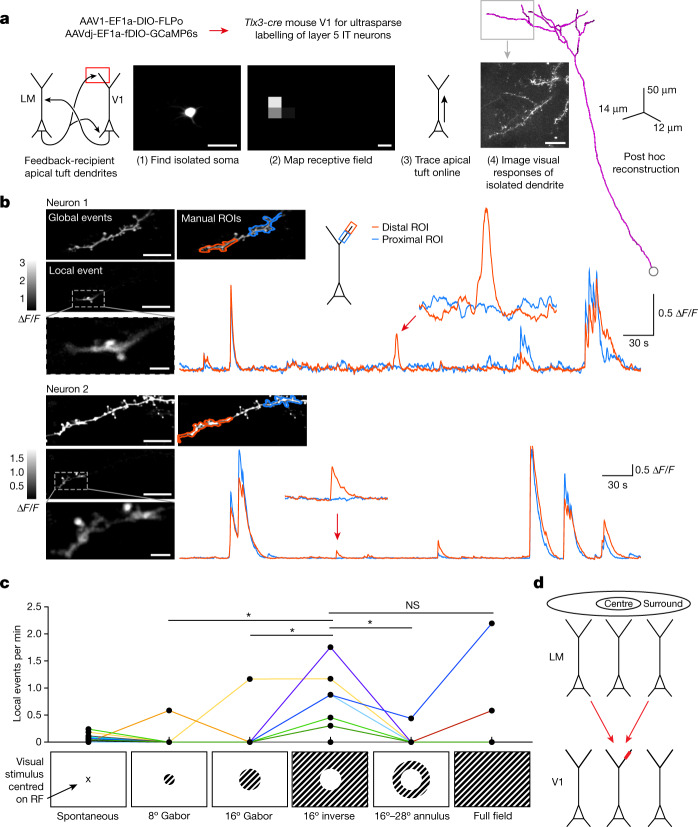
Visual stimuli that recruit facilitating feedback drive local calcium events in apical tuft dendrites. **a**, Experimental design. Ultrasparse expression within layer 5 IT neurons defined by Cre expression was achieved by combining AAV-mediated Cre-dependent FLP expression with AAV-mediated FLP-dependent GCaMP6s expression. Isolated somata were identified using two-photon imaging, their receptive fields were mapped and apical dendrites were traced. Visual stimuli were then positioned relative to the receptive field and the visual responses of the identified dendrites were imaged. Scale bars, 50 μm (left), 8° (middle) and 25 μm (right). **b**, Example local dendritic events from two different neurons. For each neuron, top left, mean dendritic segment fluorescence during global events. Middle left, mean fluorescence during local dendritic event. Bottom left, local dendritic event magnified. Note that at least two spines were simultaneously active along with a limited extent of the adjacent branch. Top right, manually drawn ROIs, and illustration of their location on an idealized pyramidal cell morphology. Inset: magnification of an independent event. Scale bars, 10 μm (neuron 1 and 2, top and middle) and 2 μm (neuron 1 and 2, bottom). **c**, Stimulus-dependence of independent events showing that the inverse stimulus, which provides the most effective stimulation of the surround, drives the most independent events. Results from *n* = 13 branches belonging to 9 neurons. The lines connect responses obtained from a single branch (17 trials per minute). Kruskal–Wallis test across all stimuli (*P* = 0.01) followed by post hoc Dunn’s test: 8° versus inverse (*P* = 0.041), 16° versus inverse (*P* = 0.047), inverse versus annulus (*P* = 0.034) and inverse versus full field (*P* = 0.48). Stimuli are schematized as disks to illustrate size and shape, presented experimentally as sinusoidal gratings with Gaussian masks and no sharp edges. RF, receptive field. **d**, Schematic illustrating topographically offset feedback facilitating events in apical tuft dendrites.

**Extended Data Fig. 4:**
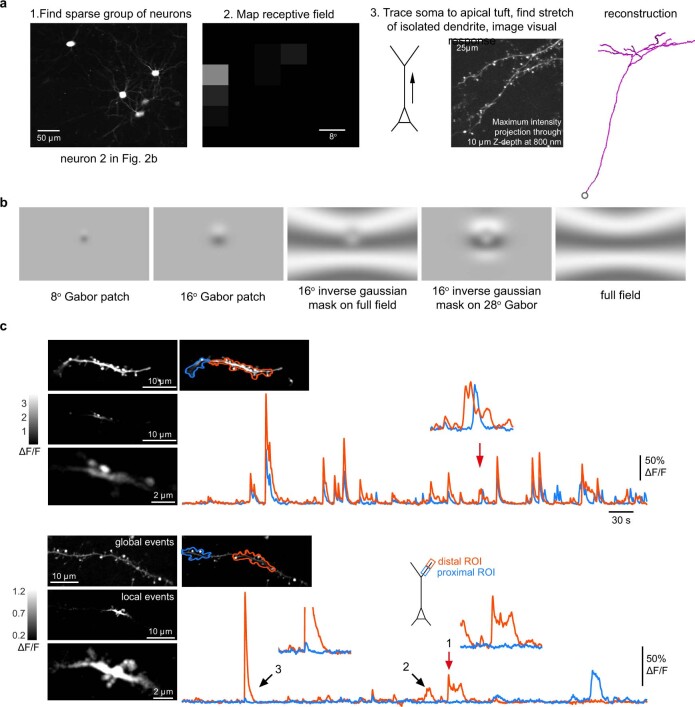
Apical tuft dendrite imaging experiments. **a**, Example illustrating experimental workflow, which consisted of finding a sparsely labelled group of neurons, mapping visual receptive fields, tracing from the soma to the apical tuft while imaging at 800 nm and then finding a branch that was relatively uninterrupted by other processes before displaying visual stimuli relative to the known receptive field. **b**, Visual stimuli used in this experiment. **c**, Two additional examples of dendrites that exhibited independent events. Bottom example illustrates challenges to automated detection. Event #1 was included in our data as an independent event that clearly involved at least two distinct spines and the branch. Event #2 was similar to #1, but only one spine and the branch were visibly activated during this smaller magnitude event. Event #3 was a global event that happened to be larger magnitude in the distal ROI in comparison to the proximal ROI.

To test our prediction, we developed a dual-recombinase approach for ultrasparse labelling of layer 5 IT neurons that enabled us to measure calcium signals from fine distal tuft dendrites of individual neurons with minimal contamination from other sources (Fig. 3a). This strategy allowed us to trace fine apical tuft dendrites to their parent somata, map their receptive fields and, finally, image the tuft dendrites at a high magnification during visual stimulation (Fig. 3b and Extended Data Fig. 4a). To test our prediction, we displayed sinusoidal gratings shaped with Gaussian masks to cover the centre of the receptive field, the surround or various combinations of both, without matching somatic orientation preference (Fig. 3c and Extended Data Fig. 4b).

**Extended Data Fig. 5:**
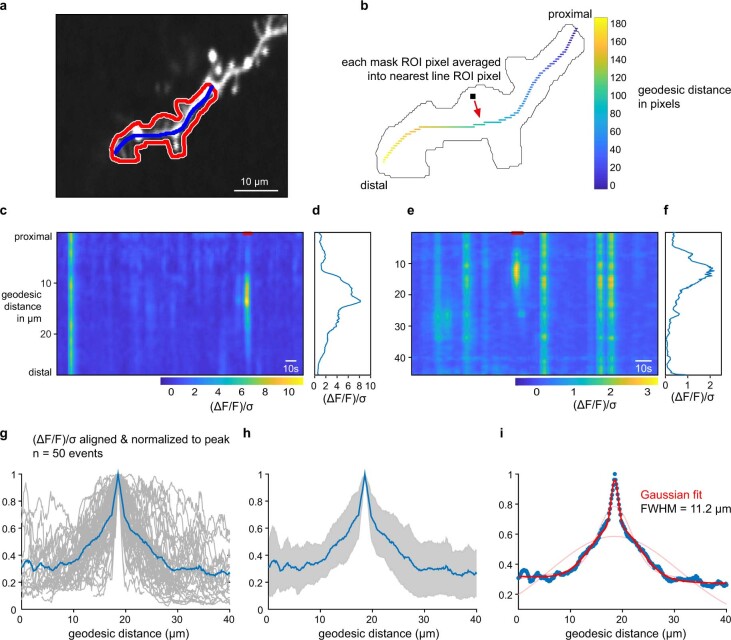
Spatial scale of local events in apical tuft dendrites. **a**, Two separate ROIs were drawn over each dendritic segment where events were identified. One “mask” ROI encapsulated the entire branch with its spines, and the other “line” ROI traced a single pixel wide line along the branch itself. **b**, Fluorescence measured in each pixel in the mask ROI was averaged into the nearest pixel of the line ROI, along with all other mask ROI pixels for which that line ROI pixel was the nearest. The geodesic distance between each line ROI pixel and the most proximal line ROI pixel was measured. **c**, Fluorescence over time and geodesic distance along an example dendrite. The mean fluorescence in each resulting geodesic distance pixel was smoothed with a 4 s moving average and then converted to (F-F_0_)/F_0_, where F_0_ was assigned as the 10^th^ percentile of the fluorescence in a running window 45 s wide. We then normalized this ΔF/F by the standard deviation of the whole trace to facilitate comparisons across experiments. **d**, The spatial profile of fluorescence over geodesic distance averaged over the frames identified with red band in c. **e**, As in c for another example neuron. **f**, As in d for another example neuron. **g**, The spatial profile of all identified events, aligned by their peak location and normalized to that value in grey, their average in blue. **h**, Same data as in g, average ± s.d. **i**, Average spatial profile is well fit by a 3-term Gaussian with full-width at half-maximum of 11.2 μm.

We found that visual stimuli could evoke local dendritic calcium transients that were spatially more extensive than single spine events, but not as extensive as global calcium events. These involved at least two spines and a limited stretch of the dendritic branch simultaneously activated, while the same branch was not activated proximally (closer to the soma; Fig. 3b, Extended Data Fig. 4c and Supplementary Videos 1 and 2). These events were localized in comparison to global events, which involved activation of the entire dendritic branch^25^ (Supplementary Video 2). We found 50 such events in 13 branches belonging to 9 neurons out of a total of 24 branches belonging to 13 neurons imaged. The spatial spread of these local events was variable, but the average event had a full-width at half maximum of 11.2 µm (Extended Data Fig. 5), similar to the spatial extent of pharmacologically identified NMDA spikes described in vitro^7,22,26^. Importantly, the frequency of these events was modulated by visual stimulus type—they occurred most commonly during the presentation of the ‘inverse’ stimulus (Fig. 3c). These results indicate that surround stimuli, which should recruit relatively more facilitating feedback from LM, also recruit NMDA-spike-like localized calcium events in tuft dendrites. Functionally, this means that information pertaining to sensory context is locally and nonlinearly integrated in apical dendrites in V1. By contrast, a visual stimulus inside the receptive field produced almost no local events, but did produce global events, which we examined next.

**Extended Data Fig. 6:**
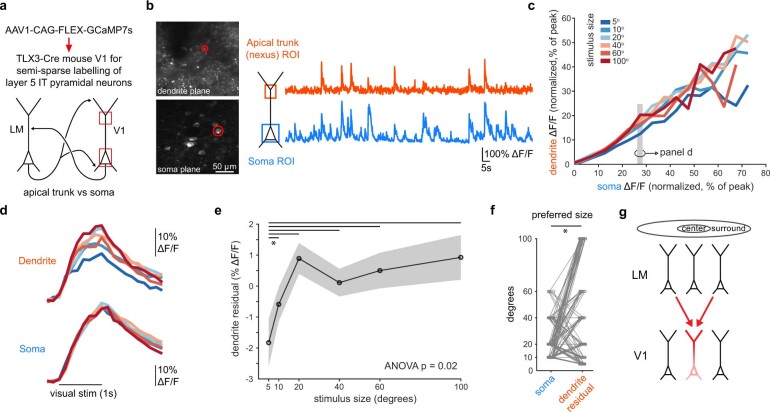
Visual stimuli that recruit enhanced facilitating feedback preferentially drive dendritic activity. **a**, Semi-sparse expression allows tracing of apical trunks to their parent somata. **b**, Left, soma and dendrite regions-of-interest. Right, fluorescence from a connected soma and apical dendrite. **c**, Population average dendritic fluorescence as a function of somatic fluorescence, for six stimulus sizes. Trials sorted by somatic activity level, binned in 5% increments, averaged across cells (n = 57 neurons with receptive fields <20° from stimulus centre, and size tuning (p < 0.01) with preference for 20° gratings). Two-way ANOVA on population data to determine effect of somatic activity, stimulus size, and their interaction on dendritic fluorescence showed a significant effect of stimulus size, F(5,131) = 5.96, p = 5 x 10^−5^, and a significant interaction, F(5,131) = 4.74, p = 5 x 10^−4^. **d**, Population average somatic and dendritic calcium fluorescence traces for different stimuli from one somatic activity bin, indicated in panel c. **e**, Dendritic fluorescence as a function of stimulus size, averaged across neurons after effect of somatic fluorescence is removed on a cell-by-cell basis (residual after subtraction of fit to somatic fluorescence; Methods). One way repeated measures ANOVA on n = 57 neurons shows significant effect of size, F(5,280) = 2.681, p = 0.02. Post-hoc tests show residual at 5° is significantly smaller than residuals at larger stimulus sizes (paired t-tests, p < 0.05). **f**, Dendritic and somatic size preference. Dendrites prefer larger stimuli as a population (paired t: p = 1 x 10^−6^). Data from n = 131 neurons with receptive fields <20° from stimulus centre, significant size tuning (p < 0.01), and preferring 10°,20°, 40° or 60° gratings. Data from 26 sessions in 9 mice. Dendritic preference computed from residuals as in e. **g**, Schematic illustrating topographically offset feedback facilitating dendritic excitation during global events.

**Extended Data Fig. 7:**
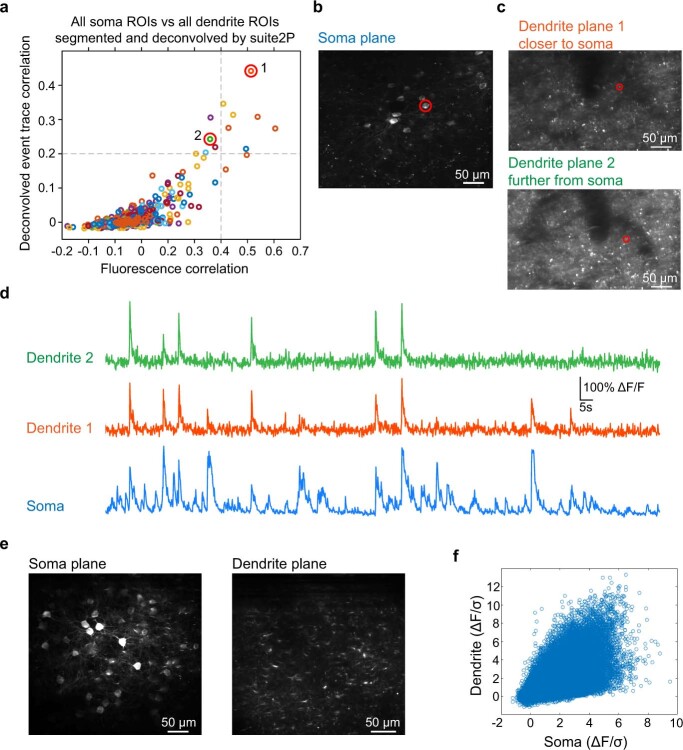
Identifying connected soma-apical dendrite pairs with semi-sparse expression. **a**, Correlation between the fluorescence traces (X) and between the deconvolved event traces (Y) for all possible pairs of somata and dendrites from one session. Dashed line: thresholds used to identify connected pairs. **b**, Soma plane from this example session. **c**, Two dendrite planes from same example session. **d**, Calcium signals in the soma and dendrites highlighted by red circles. Both dendrites belong to circled soma, but only one pair makes it past the conservative thresholds. **e**, Another example that illustrates variability in expression density. Denser end of the range we obtain still allows identification of apical trunk and nexus below layer 1. **f**, Dendrite fluorescence as a function of soma fluorescence, n = 76 neurons (receptive field <10 degrees of stimulus centre, preferring 10, 20, 40 or 60 degrees). Fluorescence was baseline-subtracted (F_0_ = 10^th^ percentile in a 1 min running window) normalized by its standard deviation over the whole recording duration and decimated to 1 Hz for this plot. Note that high fluorescence in the dendrite in the absence of high fluorescence in the soma is not a feature of the data.

**Extended Data Fig. 8:**
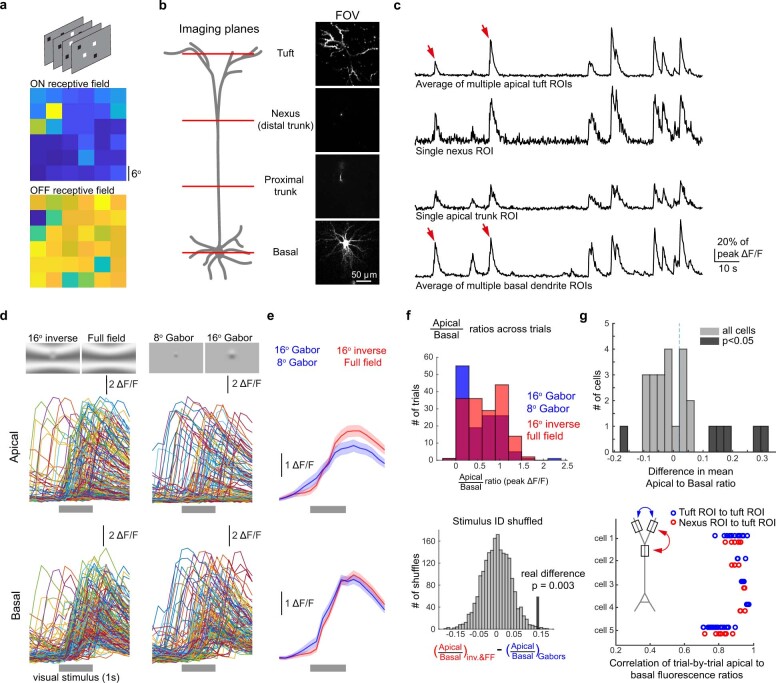
Modulation of global events in apical dendrites relative to basal dendrites by visual stimuli. **a**, Receptive field mapping with sparse noise stimuli and example receptive field. **b**, Left, schematic with volume imaging planes. Right, example neuron showing ultra-sparse expression. Isolated somata were identified, their receptive fields mapped, and apical dendrites traced. Visual stimuli (same as in Fig. 3) were then positioned relative to the receptive field. **c**, Traces from different dendritic locations. Arrows highlight events with variable ratio of apical to basal fluorescence. **d**, Top left, apical dendritic responses to inverse and full field stimuli. Bottom left, basal dendritic responses to same stimuli. Top right, apical dendritic responses to Gabor stimuli. Bottom right, basal dendritic responses to same stimuli. Each trace shows one trial, with fluorescence from all apical and all basal ROIs averaged to get one trace per compartment. **e**, Same data as in d, averaged across trials (mean ± s.e.m.) with responses to Gabor stimuli in blue and responses to inverse and full field stimuli in red, separately for apical (top) and basal dendrites (bottom). **f**, Top, distribution of apical-to-basal fluorescence ratios across trials for the two stimulus classes for one cell. Bottom, same data, comparing the real difference between the apical-to-basal fluorescence ratios (difference of means of histograms above) to the shuffling stimulus ID for the same trials. **g**, Top, apical-to-basal ratio differences for 26 recordings. Significantly modulated neurons shown in dark grey. Dashed line shows mean of all cells. Bottom, for the five significantly positively modulated neurons recorded, the apical-to-basal ratio was computed individually for each apical ROI against the average of all basal ROIs. The correlation between the apical-to-basal ratios across apical ROIs, with correlation of trunk and tuft ROIs in red and the correlation of tuft ROIs in blue.

Somatic activity is often associated with global events that engage the entire apical tuft^25^. Such global dendritic events could arise due to backpropagating action potentials (bAPs), dendritic calcium spikes or a combination of both^21,27^. To assess whether global dendritic events carry a similar signature of surround facilitation as local events, we imaged somata and apical dendrites expressing GCaMP7s semi-sparsely, while presenting Gabor patches of increasing size as visual stimuli, which contain increasingly higher surround energy (Extended Data Figs. 6a,b and 7). Focusing on a population of cells preferring 20° Gabors, we found that 53% (*n* = 30 out of 57) of cells individually showed a significant effect of visual stimulus size on dendritic activity after controlling for somatic activity (one-way analysis of variance (ANOVA) on linear regression residuals, *P* < 0.05; Methods and Extended Data Fig. 6c–e). Residual dendritic signals increased as stimulus size increased to the preferred size. Beyond the preferred size, somata were suppressed, but residual dendritic signals remained elevated on average. Across a larger population of neurons with diverse size preference (*n* = 131), dendritic residuals were biased to prefer larger visual stimuli than their parent somata (Extended Data Fig. 6f,g). In a second experiment, we used the same stimuli as those in Fig. 3 and measured the responses of apical dendrites relative to basal dendrites in individual neurons expressing GCaMP6s ultrasparsely, finding a similar preference of the apical compartment for stimuli with more surround energy (Extended Data Fig. 8). Thus, visual stimuli with more surround energy recruit the apical dendritic compartment relatively more than the rest of the neuron during global events, similar to the conditions that drove local dendritic events. This increase in apical dendritic recruitment may reflect calcium events of dendritic origin or a modulation of bAP efficacy. In summary, dendritic events in V1 layer 5 pyramidal cells involving calcium entry with spatial scales ranging from local to global are all modulated by sensory stimuli with a preference for surround stimulation. This suggests that feedback connectivity and dendritic recruitment are co-organized—they obey a similar centre–surround organization.

## Feedback contributes to dendritic signals

**Fig. 4:**
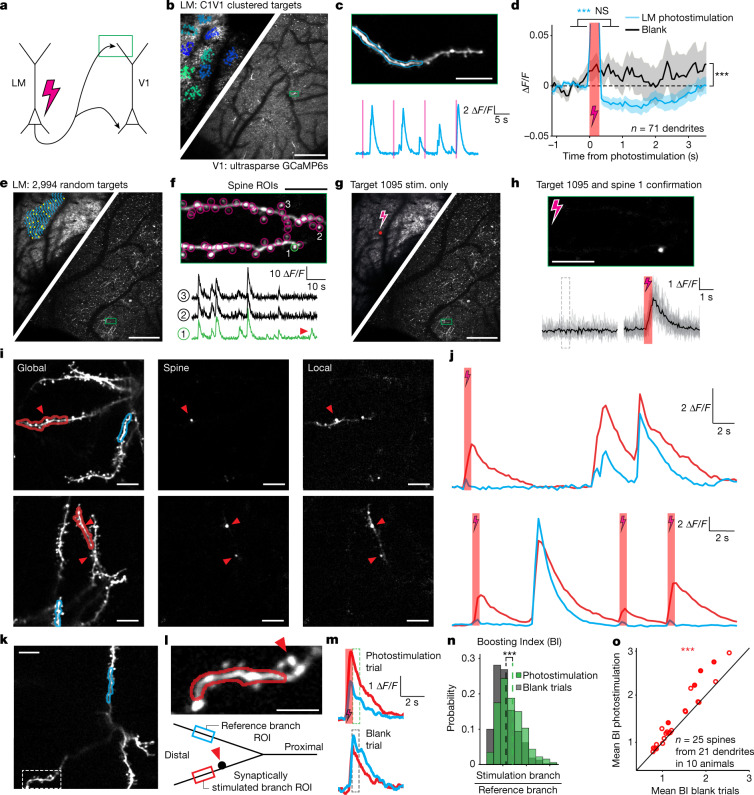
LM feedback drives branch-specific local dendritic calcium signals in V1. **a**, Two-photon optogenetic stimulation of LM neurons during simultaneous two-photon imaging of apical dendrites in V1. **b**, Left, opsin expression and target groups in LM for clustered stimulation. Right, sparse GCaMP expression in V1. **c**, Top, example dendrite. Bottom, fluorescence from the blue ROI. Photostimulation is indicated by the red lines. **d**, Average photostimulation response (blue) and average blank trial (black). *n* = 71 dendrites in 14 animals. Statistical analysis was performed using signed-rank tests; *P* = 1.1 × 10^−7^ (before versus after stimulation), *P* = 0.74 (before versus after blank), *P* = 3.2 × 10^−6^ (stimulation versus blank). **e**, Grid of targets for random stimulation; one group is shown in yellow. **f**, Top, example dendrite and spine ROIs. Bottom, fluorescence from three ROIs. The arrowhead indicates an independent spine event. **g**, Testing a putative connection (target 1095 to spine 1) after it was identified through analysis of independent events. Stim., stimulation. **h**, Isolated stimulation confirms connection. Top, photostimulus-triggered average image showing responsive spine. Bottom, blank and photostimulus responses from spine. **i**, Two examples of photostimulation-triggered local events. Left, global event showing stimulated and reference branch ROIs. Middle, responsive spines. Right, a single trial showing a branch-specific event. **j**, Fluorescence from ROIs in **i**. **k**, Example FOV for the boosting analysis showing an ROI on a reference branch; the box indicates a branch carrying a feedback-recipient spine. **l**, Top, higher-magnification image showing a feedback-recipient spine (arrowhead) and an ROI excluding it. Bottom, schematic showing layout of ROIs for the boosting analysis. **m**, Top, example photostimulation trial showing fluorescence from the synaptically stimulated branch and reference branch. Bottom, the same for a blank trial. **n**, Distributions of boosting indices for stimulation and blank trials from one recording with significant boosting. Statistical analysis was performed using a rank-sum test; *P* = 1.16 × 10^−8^. The dashed lines indicate the mean values. **o**, Mean boosting indices (BI) for photostimulation trials are higher than for blank trials across recordings. Statistical analysis was performed using a paired *t*-test; *P* = 0.0002. The filled circles indicate individually significantly modulated neurons (*P* < 0.05). Scale bars, 400 μm (**b**,**e** and **g**), 20 μm (**c** (top), **f** (top), **h** (top), **i** and **k**), 10 μm (**l**).

To establish a causal relationship between functional connectivity and dendritic recruitment, it is necessary to show that feedback from LM can drive nonlinear dendritic integration in apical dendrites. To test this, we combined two-photon optogenetic stimulation of LM neurons with simultaneous apical dendritic imaging in V1 in two sets of experiments (Fig. 4a) in which we expressed soma-restricted C1V1 in LM, and GCaMP6s ultrasparsely in V1. First, stimulating clustered groups of LM neurons (25 targets per cluster) while imaging apical dendritic segments in V1 (Fig. 4b,c) reduced the average calcium signals measured from apical dendrites (Fig. 4d). Random 20% subsamples of all stimulation group–dendrite pairs consistently yielded suppression of calcium signals, with only 0.7% of subsamples producing an average response above zero. This suggests that feedback suppression mediated by LM is dense and non-specific, whereas feedback facilitation may be sparse.

**Extended Data Fig. 9:**
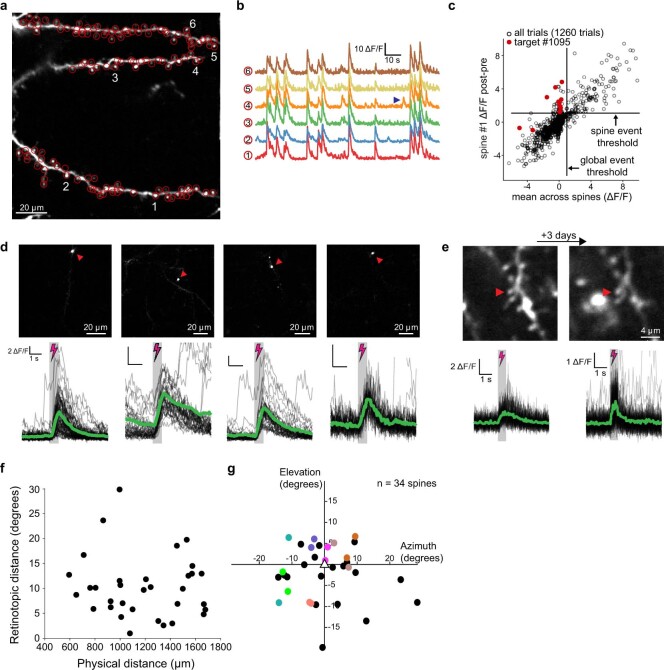
Two-photon photostimulation-triggered spine mapping. **a**, Example dendrite and ROIs used for spine fluorescence extraction. Same experiment as shown in Fig. 4, where panel f shows a section of the FOV shown here at higher magnification. **b**, Fluorescence from six example ROIs. **c**, Photostimulation responses of example ROI #1 plotted against the mean photostimulation response across all spine ROIs. Trials that produced independent activity were identified as passing a threshold applied to signals from that spine and not passing a threshold applied to the average spine. Targets active on those trials, like the example target #1095 shown, were identified. **d**, Four example spines from four recordings. Top, stimulation-triggered average image showing independently activated spines. Bottom, the stimulation triggered fluorescence traces from those example spines. **e**, An example spine and stimulation target pair that were identified in two separate experimental sessions, 3 days apart. **f**, Distribution of retinotopic and physical distances between effective target in LM and the parent soma of the responsive spine. Retinotopic distances were measured using widefield intrinsic imaging under anaesthesia and temporal retinotopic mapping (Kalatsky et al., 2003). **g**, Retinotopic position of effective targets in LM plotted relative to the retinotopic position of the parent soma of the responsive spine, positioned at the origin.

Next, to identify potentially rare facilitating connections and stimulate them in isolation, we sought to increase the number of potential connections that we assayed in a single experiment. To this end, we stimulated random groups of 8–25 targets drawn from a three-dimensional (3D) target grid in LM composed of thousands of targets and recorded the responses of all of the visible spines on apical dendrites that we imaged in V1 (Fig. 4e,f). In these experiments, opsin expression was either limited to TLX^+^ neurons or also included layer 2/3 pyramidal neurons to increase the number of potential presynaptic partners. Using online analysis of stimulation-triggered spine signals, we identified potentially connected pairs of optogenetic targets and responsive spines (Extended Data Fig. 9a–c). We then performed targeted stimulation of the putative presynaptic neurons either individually or in small groups to validate individual connections (Fig. 4g). Remarkably—given the considerable distance between targets in LM and the recipient neurons in V1, as well as the challenge of finding the relevant spines on the postsynaptic pyramidal cell being imaged—we were able to confirm a substantial number of spines as unambiguously responsive to stimulation of individual targets in LM (Fig. 4h and Extended Data Fig. 9d–g; signed-rank test, before versus after stimulation, *P* < 0.01; 34 significantly responsive spines in 26 neurons in 11 animals out of 147 recordings in 25 animals). Detection of spines active in isolation (Supplementary Video 3) confirmed that the signals were driven by synaptic input from LM. These results directly identify the presynaptic source and postsynaptic targets of LM inputs on the apical dendrites of layer 5 pyramidal cells in V1, and indicate that feedback excitation mediated by LM is sparse.

**Extended Data Fig. 10:**
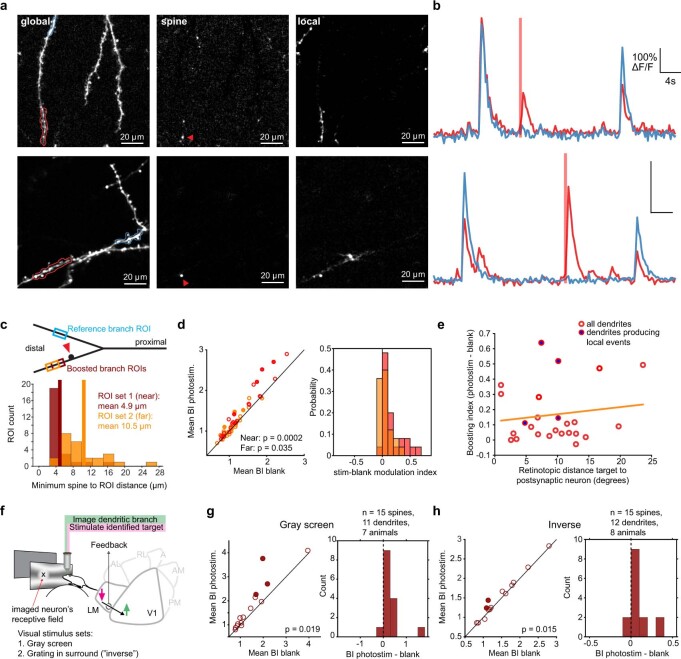
Branch-specific boosting analysis. **a**, Two additional examples of branch-specific local events in response to photostimulation of identified presynaptic targets in LM. Left, average image of global events, showing stimulated (incl. spine) and reference branch ROIs. Middle, spines responsive to feedback. Right, average image of single trial showing branch-specific event. **b**, Fluorescence extracted from those ROIs, showing the variability of stimulated vs reference branch signal strength and the branch-specific events driven by photostimulation, indicated by red bars. **c**, Top, schematic illustrating ideal layout of ROIs used for boosting analysis. Bottom, distribution of minimum distances between stimulated branch ROIs and the identified feedback-recipient spine. **d**, Mean boosting indices for blank vs photostimulation trials. Each recording appears once for “near” ROIs and once for “far”. “Near” ROIs same as reported in Fig. 4. Filled circles are individually significant neurons (t-test, p < 0.05). Right, same data plotted as a histogram of differences between boosting indices measured during stimulation and during blank trials. **e**, Boosting index (difference between photostimulation and blank, same data as in Fig. 4) as a function of retinotopic distance between target location and parent soma location. **f**, Schematic illustrating visual stimulation delivered simultaneously with feedback stimulation. **g**, Boosting indices measured from Near ROIs while the visual stimulus was a grey screen. Left, scatter plot of indices measured on blank vs on stimulation trials. Right, same data shown as a histogram of differences between trial types. **h**, Boosting indices measured from Near ROIs while the visual stimulus was an inverse grating positioned around the receptive field of the V1 neuron. Left, scatter plot of indices measured on blank vs on stimulation trials. Right, same data shown as a histogram of differences between trial types.

Having identified excitatory connections between LM neurons and V1 apical dendrites, we next examined whether this excitatory feedback can recruit local dendritic calcium signals. Notably, in the apical tuft of some V1 layer 5 pyramidal neurons, LM stimulation triggered local dendritic calcium events that resembled the visually evoked local events shown in Fig. 3 (4 out of 26 neurons, on one to five trials per neuron; Fig. 4i,j, Extended Data Fig. 10a,b and Supplementary Video 4). This suggests that feedback input from LM is capable of triggering local dendritic nonlinear events in the apical tuft of V1 pyramidal neurons. Next, to provide further support for the recruitment of dendritic nonlinearities by feedback, we examined whether LM inputs can boost calcium signals in the recipient dendritic branch. We identified a spine activated by LM input, and quantified the photostimulation-induced modulation of neighbouring dendritic calcium signals in that branch in comparison to calcium signals recorded from a sister branch (Fig. 4k–n). We placed a target region of interest (ROI) distally on the branch containing the feedback-recipient spine, but excluding the spine itself (mean minimum spine-to-ROI distance across cells, 4.9 ± 0.57 µm (mean ± s.e.m.); Fig. 4l). We placed another reference ROI for comparison on a different branch belonging to the same neuron, or occasionally more proximally on the same branch (ROIs identified for 25 out of 34 spines) and calculated the ratio of calcium signals from the stimulated branch compared with that of the reference (the boosting index; Fig. 4m,n). We compared the boosting index computed for feedback photostimulation trials with the boosting index computed for blank trials lacking photostimulation (Fig. 4o). We found that photostimulation of LM inputs preferentially enhanced calcium signals in the branch containing the activated spine relative to the reference branch (increase in boosting index, 10.8 ± 2.2% (mean ± s.e.m.); *P* = 0.0002; Fig. 4o). Repeating this analysis with the target ROI moved further away from the feedback-recipient spine (minimum spine-to-ROI distance, 10.5 ± 1.05 µm (mean ± s.e.m.)) produced the same result, conservatively showing that this effect is spatially extended beyond the spine itself (Extended Data Fig. 10c,d). This effect did not depend on retinotopic distance and was present regardless of whether feedback photostimulation was performed simultaneously with visual stimulation or on its own (Extended Data Fig. 10e–h). Thus, we provide causal evidence that LM input can trigger local dendritic calcium signals similar to those driven by surround visual stimuli, and can also drive branch-specific boosting of ongoing spontaneous and visually evoked activity.

Taken together, these results reveal several features of feedback. First, feedback suppression is dense, arising from many LM neurons. Second, feedback facilitation is sparse, arising from few LM neurons. Third, feedback can drive dendritic branch-specific local calcium signals that are spatially extended beyond individual feedback-recipient spines. Finally, feedback-driven dendritic calcium signals can be large enough to account for local dendritic calcium events driven by visual stimuli. Overall, these results establish a causal connection between LM feedback and apical dendritic calcium signals reflecting nonlinear postsynaptic integration.

## Behaviour regulates feedback and dendrites

**Extended Data Fig. 11:**
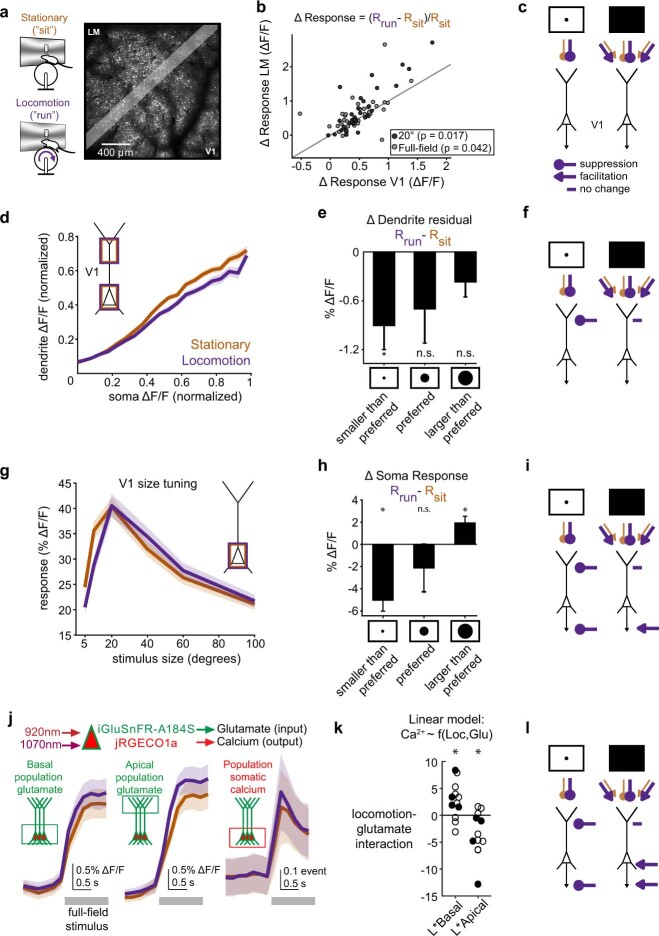
Locomotion regulates feedback and dendritic excitability. **a**, FOV for measuring modulation of visual responses in V1 and LM. **b**, Locomotion facilitates visual responses more in LM than V1 (signed-rank test, 20°: p = 0.017, full-field: p = 0.042). **c**, Increased feedback predicts increased suppression for smaller stimuli, and increased suppression and facilitation for larger stimuli. **d**, Dendritic fluorescence as a function of somatic fluorescence, during locomotion and stationarity (mean ± sem, n = 131 neurons). A 2-way ANOVA to determine effect of somatic fluorescence and locomotion on dendritic fluorescence shows significant interaction: F(1,48) = 8.86, p = 0.0046. **e**, Change in dendritic response with locomotion (mean ± sem). For each neuron we removed the effect of somatic activity and categorized stimuli as smaller than preferred (paired t-test, p = 0.003,) preferred (p = 0.112), larger than preferred (p = 0.067). **f**, Apical dendrites are suppressed by locomotion when small stimuli, but not larger stimuli, are presented. **g**, Somatic size tuning during locomotion and stationarity (n = 131). **h**, Somatic response modulation. (mean ± s.e.m., smaller than preferred: paired t-test, p = 5.45x10^−7^, preferred: p = 0.32, larger than preferred: p = 0.002). **i**, Locomotion reduces somatic response to small stimuli but enhances response to large stimuli. **j**, Top, strategy for dual-colour input-output imaging. Bottom, stimulus-triggered average glutamate and calcium fluorescence during stationarity and locomotion (n = 12 sessions, 4 mice). **k**, Interaction coefficients between locomotion and apical/basal glutamate from linear model (Methods). Basal coefficients are significantly above (t-test, p = 0.01), apical coefficients below zero (p = 0.02). Filled circles are sessions with individually significant effect of omitting interactions. **l**, Locomotion-induced enhancement of feedback, suppression of apical dendrites and somata during presentation of smaller stimuli, and enhancement of basal inputs and somata for larger stimuli.

**Extended Data Fig. 12:**
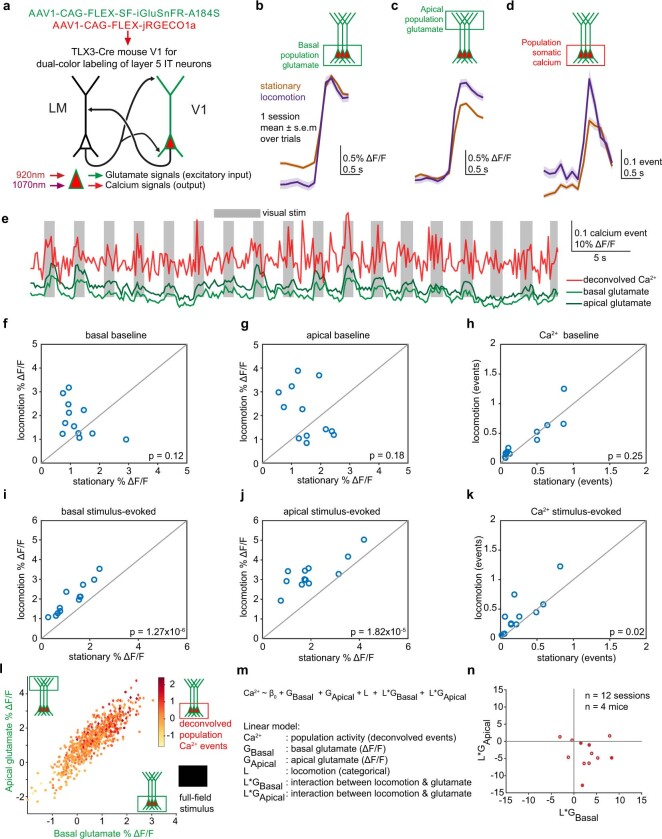
Locomotion-induced changes in glutamate, calcium signals and their relationships. **a**, Schematic illustrating dual-colour expression strategy. **b**, Basal glutamate fluorescence over entire FOV during stationary and locomotion periods, averaged over all trials in one session. **c**, Apical glutamate fluorescence over entire FOV during stationary and locomotion periods, averaged over all trials in one session. **d**, Population activity quantified as deconvolved events across all ROIs over entire FOV during stationary and locomotion periods, averaged over all trials in one session. **e**, Raw traces of iGluSnFR fluorescence and deconvolved calcium signals summed across the population. Grey bars are visual stimulus. **f**, iGlu-SnFR fluorescence in basal dendrites during the baseline period did not change with locomotion (n = 12 sessions in n = 4 mice, t-test, p = 0.12). **g**, iGlu-SnFR fluorescence in apical dendrites during the baseline period did not change with locomotion (t-test, p = 0.18). **h**, Deconvolved events during the baseline period did not change with locomotion (t-test, p = 0.25). **i**, Stimulus evoked change in mean iGlu-SnFR fluorescence in the basal dendrite planes increased with locomotion (t-test, p = 1.3 x 10^−6^). **j**, Stimulus evoked change in mean iGlu-SnFR fluorescence in the apical dendrite planes increased with locomotion (t-test, p = 1.8 x 10^−5^). **k**, Stimulus evoked change in deconvolved events increased with locomotion (t-test, p = 0.02). **l**, Three dimensional population level input-output function for one session. **m**, Linear model fit to each session in order to measure the interaction between locomotion and basal and apical glutamate to population output gain. **n**, Coefficients of the interaction show that locomotion and basal glutamate interact positively (t-test, p = 0.01), while locomotion and apical gain interact negatively (t-test, p = 0.02). Solid data points are sessions with individually significant interactions between running and glutamate (Wald test, p < 0.05, Methods).

**Extended Data Fig. 13:**
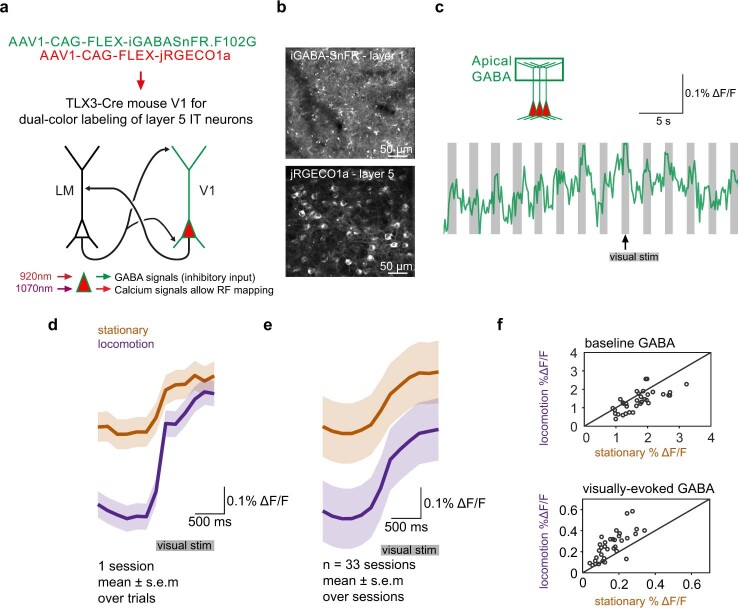
Locomotion suppresses spontaneous GABAergic signalling in layer 1 neurons but enhances sensory-evoked GABAergic signals. **a**, Expression and excitation strategy for dual-colour imaging. **b**, Average iGABA-SnFR fluorescence (top) and jRGECO1a fluorescence (bottom) in a typical field-of-view (300 μm). Visual stimuli were 20° or full field sinusoidal gratings and they produced qualitatively similar results. **c**, Example raw trace of iGABA-SnFR fluorescence recorded over the entire field of view and averaged over two imaging planes in layer 1. **d**, Mean GABA fluorescence over entire field-of-view during stationary and locomotion periods, averaged over trials in one session. **e**, Mean GABA fluorescence over entire field-of-view during stationary and locomotion periods, averaged across n = 33 sessions. **f**, Mean fluorescence during stationarity vs locomotion. Top, GABA fluorescence during baseline periods significantly decreased with locomotion (t-test, p = 5.5 x 10^−5^). Bottom, stimulus evoked (baseline-subtracted) GABA fluorescence significantly increased with locomotion (t-test, p = 3 x 10^−7^).

If feedback and dendritic recruitment are causally related, they may also be co-regulated by learning^28,29^, behaviour^3,6^ or, on shorter timescales, by moment-to-moment variations in behavioural state^25^. To examine whether behavioural state regulates feedback and dendrites, we focused on locomotion^30,31^ and found that it enhances visual responses in layer 5 of LM (Extended Data Fig. 11a,b), similar to findings in layer 2/3 of LM^32^. Predicting that increased activity in LM might lead to increased feedback facilitation as well as inhibition of apical dendrites in V1, we measured glutamate and GABA signals in layer 1 of V1 using the genetically encoded sensors iGluSnFR-A184S (Methods and Extended Data Fig. 12) and iGABA-SnFR (Methods and Extended Data Fig. 13), respectively, and found that locomotion enhanced both signals. Given the increased activity in LM and increased input to apical dendrites associated with locomotion, we next looked for changes in dendritic calcium signals in V1. We found that apical dendritic activity was suppressed by locomotion during the presentation of visual stimuli smaller than the neurons’ preferred size, while it did not change for larger stimuli (Extended Data Fig. 11d–f). Locomotion also reduced somatic responses to small stimuli, while enhancing responses to larger visual stimuli (as observed previously^31^; Extended Data Fig. 11g–i). A reduction in somatic and dendritic responses to small stimuli is consistent with increased feedback suppression of apical dendrites. However, the locomotion-induced somatic enhancement in response to larger stimuli is incompatible with the lack of apical dendritic enhancement for larger stimulus sizes and must instead be mediated by basal dendrites. To resolve this, we performed a dual-colour two-photon input–output imaging experiment, measuring basal and apical glutamatergic inputs and output activity using the red calcium indicator jRGECO1a (Methods and Extended Data Figs. 11j and 12). A linear fit to the relationship of dendritic inputs to population output indicated that, during locomotion, apical inputs become less effective, whereas basal inputs become more effective, potentially supporting the enhancement of responses to larger stimuli (Extended Data Figs. 11k,l and 12l–n). This enhancement may involve changes in basal dendritic excitability^26^ as well as enhanced thalamic responses^33^, and an NDNF-interneuron-mediated shift in inhibition from the soma to apical dendrites^34^. Collectively, these results are consistent with our framework of feedback modulation of dendritic excitability. In summary, while locomotion involves diverse and distributed changes throughout the brain, our results suggest that moment-to-moment changes in dendritic excitability linked to modulation of feedback may be a contributing mechanism.

## Discussion

Our experiments reveal findings about the organization of cortical feedback, nonlinear dendritic integration and their relationship in vivo. First, we developed a powerful all-optical strategy for investigating the functional connectivity between brain areas at cellular resolution, which revealed a retinotopic organization of feedback in visual cortex—LM is relatively more suppressive of V1 when connections are between regions responsive to the same part of visual space. Conversely, LM is relatively more facilitating of V1 when connections are between regions responsive to more distant parts of visual space. This ‘suppressive centre, facilitating surround’ organization contrasts with feedback from the frontal cortex—projections from cingulate cortex exhibit a facilitating centre and suppressive surround in V1^35^. This difference may reflect distinct functions of feedback from different sources—while feedback from frontal cortex may support attentional modulation^35^, our results are more consistent with models of predictive coding^36,37^. Feedback from higher visual areas has been modelled using subtractive elements carrying predictions^38^. We provide mechanistic support for such models by validating one of their tenets: feedback aligned to the feedforward hierarchy should be suppressive^36^ (consistent with silencing experiments^15,16^ and corticothalamic feedback^39^). Identifying the inhibitory circuits that mediate feedback suppression will be crucial. Our demonstration of a ‘facilitating surround’ in turn provides a circuit mechanism for cooperative interactions between stimuli and their sensory context^40,41^ as well as for excitatory responses to surround-only visual stimuli^17,42^. Silencing LM reduces V1 responses to surround-only visual stimuli^17^, which is consistent with our results on LM to V1 functional connectivity, and our demonstration that LM inputs can engage dendritic nonlinearities in V1. The generality of these results may be constrained by technical factors such as the limited temporal resolution of GCaMP, which may obscure faster modulations of feedback^43^. Similarly, functional connectivity may depend on visual contrast, feature selectivity, cell types, cortical layer and source area. It will be crucial to understand these dependencies and identify the synaptic wiring diagram that underlies functional connectivity.

To understand how feedback works, it is essential to reveal how it is implemented at the cellular level. By combining targeted stimulation of neurons in LM with high-resolution imaging of dendritic arbours in V1, we provide a demonstration of independent local dendritic events selectively recruited by sensory stimuli designed to enhance feedback. We then identify feedback-recipient spines and show directly that the stimulation of feedback inputs to these spines can both drive and enhance branch-specific dendritic calcium signals extending beyond the identified spines. These findings suggest that feedback from LM contributes to the generation of active dendritic events in response to visual stimuli. It will be important to determine whether LM inputs or any other specific inputs are necessary for the recruitment of active dendritic events, and whether natural patterns of feedback activity recruit them. It will also be important to confirm the underlying voltage profile, true frequency and biophysical basis of these events, which are consistent with NMDA spikes^22,26^ but could also involve other mechanisms, such as other types of dendritic spikes^5,20,21^ or intracellular calcium release^44^.

Active dendritic integration has been shown to contribute to feature selectivity^45–47^. Here we show that sensory context (visual surround) also engages nonlinear dendritic mechanisms within apical tuft dendrites. By showing that feedback inputs can trigger local events in individual dendritic branches, and can also produce branch-specific boosting of dendritic activity, our study provides crucial support for the longstanding proposal that branch-specific computations are exploited in vivo^19,23,26^. Describing dendritic recruitment rules under natural behavioural and sensory conditions will be critical to a mechanistic understanding of how feedback contributes to cortical computations such as contour integration and figure–ground segmentation^3^. Feedback may generate somatic responses directly^17^ by driving active dendritic events, or it could indirectly affect dendritic integration, as engaging dendritic nonlinearities can change input–output gain both locally^48^ and globally^24^ to make other inputs, such as long range horizontal inputs^11,49^, more effective. Such gain control may allow contour integration to rely on the cooperative interaction of feedback and horizontal excitatory connections^50^. As basal dendrites also receive feedback^4^, it will be important to understand how their excitability may also be involved in mediating feedback influence.

Finally, we find that active behavioural states are associated with increased activity in a prominent feedback source to V1, increased input onto apical dendrites and modulation of dendritic calcium signals in a manner consistent with feedback topography. Our results suggest a model in which coordinated regulation of feedback and apical dendritic excitability contributes to sculpting visual representations. As interareal feedback projections and active dendritic integration are both ubiquitous features of the neocortex, the fact that they are systematically related suggests that these mechanisms may represent general principles governing coordination among cortical areas.

## Methods

All experimental procedures were carried out under license from the UK Home Office in accordance with the UK Animals (Scientific Procedures) Act (1986).

### Mice and surgeries

Male and female *Tlx3-cre* or *Tlx3-cre;CaMKII-tTA;TITL-GCaMP6s* mice aged between seven and ten weeks were used. *Tlx3-cre* (PL56) is a GENSAT BAC transgenic and has been previously described^9,51^. The other two parental lines were obtained from The Jackson Laboratories (CaMKII-tTA, 007004, ref. ^52^, and TITL-GCaMP6s (Ai94), 024104, ref. ^53^). Four to eight hours before surgery, mice were given an injection of dexamethasone (Dexadreson, 5 mg per kg body weight at 2 mg ml^−1^)^54^. Immediately before surgery, mice were given a subcutaneous injection of buprenorphine hydrochloride (Vetergesic, 1 mg per kg body weight at 0.3 mg ml^−1^) and anaesthetized with isoflurane (5% induction, <1.5% maintenance). The scalp was removed and an aluminium or titanium headplate with an 11 mm circular opening was fixed to the skull with dental cement (Super-Bond C&B, Sun-Medical). A craniotomy was performed over the caudal-lateral cortex and the dura was carefully removed. A calibrated pipette (Drummond Scientific Company, Wiretrol II, 5-000-2005) bevelled to a sharp point and connected to a hydraulic injection system (Narishige MO-1) was used to inject virus, which was diluted in a buffer solution (20 mM Tris, 140 mM NaCl, 0.001% Pluronic F-68, pH 8.0). Virus injections were made 500 µm below the surface at 0.1 µl min^−1^. Injection locations were determined using stereotactic coordinates and blood vessel patterns. Subsequent retinotopic mapping was used to confirm intended coverage of the visual cortex. After each injection, the pipette was maintained in position for 5 min before retraction. Chronic imaging windows were constructed using a single 4 mm coverslip with small pieces of coverslip optically glued to the top side to serve as an added surface to support the dental cement. Craniotomies were sealed with cyanoacrylate glue (Vetbond, 3M) and windows were fixed in place with dental cement. The animals were allowed to recover for at least 5 days. Subsequently, the animals were acclimatized to the microscopes and Styrofoam running wheels for 2–5 sessions before experiments. The number of mice included in each experiment was as follows: two-photon stimulation and population imaging (Figs. 1 and 2): 15; two-photon stimulation and dendritic imaging: 14 (Fig. 4a–d) and 10 (Fig. 4e–o); ultrasparse dendritic imaging (Fig. 3): 5; ultrasparse dendritic volume imaging (Extended Data Fig. 8): 10; semisparse dendritic imaging (Extended Data Figs. 6, 7 and 11): 9; dual-colour glutamate imaging: 4; and dual-colour GABA imaging: 7.

### Two-photon optogenetic stimulation and population imaging

Simultaneous all-optical interrogation across LM and V1 circuits was carried out by adapting existing approaches^55–62^ using a large-FOV resonant scanning microscope (Ultima 2P plus, Bruker). Expression of calcium indicator and opsin was achieved by injecting AAV2/9-Ef1a-DIO-C1V1(t/t)-mRuby2-Kv2.1 (Selmaan Chettih, Christopher Harvey; Harvard Medical School) diluted 1:13 from a stock concentration of around 6.9 × 10^14^ genome copies (g.c.) per ml into *Tlx3-cre;CaMKII-tTA;TITL-GCaMP6s* animals. 200 nl of virus each was injected into a grid of six locations positioned about 300 µm apart over LM and V1, guided by the blood vessel patterns. Two-photon calcium imaging was performed using 920 nm light delivered from a tuneable laser (InSight X3, Spectra-Physics). Simultaneous two-photon optogenetic excitation was performed using 1,030 nm light delivered from a fixed wavelength fibre laser at a 1 MHz repetition rate (Satsuma HP2, Amplitude Systèmes). The objective used was a 16×/0.8 NA (Nikon, 32/42 sessions in 11 animals) or a 10×/0.5 NA (Thor, TL10X-2P, 10/42 sessions in 4 animals) objective, yielding FOV sizes of 1,215 or 1,920 µm respectively. Volumetric calcium imaging data were acquired using an electrically tuneable lens (ETL; Optotune) focusing four planes spaced by 30 µm (7 Hz, 16×) or two planes spaced by 50 µm (15 Hz, 10×). Power post-objective was between 50–100 mW depending on expression level and imaging depth, which was 350–450 µm below the pia. Two-photon optogenetic stimulation was performed using a programmable reflective spatial light modulator (SLM) installed in-line with the stimulation path^56,57,59,63^. The 16×/0.8 NA objective was paired with an SLM (Boulder Nonlinear Systems) with 512 × 512 pixels and 7.68 mm × 7.68 mm active area, whereas the 10×/0.5 NA objective was paired with an SLM (Meadowlark Optics) with 1,920 × 1,152 pixels and 17.6 mm × 10.7 mm active area. Phase masks were computed using the weighted Gerchberg–Saxton algorithm and loaded using Blink (Meadowlark). The SLM was calibrated to compensate for the decrease in diffraction efficiency for peripheral targets, and power per neuron kept constant at 12 mW. Imaging space to SLM space conversion was achieved by burning 3D patterns into plastic slides, taking volumetric stacks, measuring burn locations and fitting affine transformations. To increase stimulation efficiency, the centre of the SLM space was offset using galvanometers such that it was close to the centroid of the current stimulation targets. Calibrations were performed using custom software written in MATLAB^64^ (https://github.com/llerussell/SLMTransformMaker3D). Stimulation patterns consisted of multiple beamlets targeting between 6 and 14 neurons. Beamlets were scanned using galvanometers moving in spiral scan patterns (10 repeats of around 16 µm, 20 ms spiral scans at 20 Hz). Synchronization was performed as previously described^64^. FOVs over V1 and LM were determined using retinotopic maps obtained from preparatory wide-field and two-photon retinotopic mapping sessions (see below). For stimulation experiments, FOVs were relocated using blood vessel patterns and the data were affine transformed to register the field to previously obtained retinotopic maps. At the beginning of the experiment, neurons responsive to photostimulation were detected using the near automatic photoactivation response mapping (NAPARM; https://github.com/llerussell/Naparm) protocol described previously^62,64^. Stimulation clusters were then designed by randomly choosing seed neurons and finding their nearest neighbours from all photoresponsive neurons. A 200-µm-wide V1/LM border zone and the top 50 lines of the two-photon frames were excluded from consideration as targets. In each experiment, between 5 and 10 (8 ± 1.1) target clusters were stimulated for a total of 129 clusters in LM and 180 clusters in V1 in 42 sessions, 15 animals. Experiments contained 1,337 ± 142 trials (mean ± s.d.). Of these trials, 20% (in 40 out of 42 experiments) or 50% (in 2 out of 42 experiments) contained only visual stimulation composed of a 2 s stimulus and 6 s intertrial interval (V trials). The remaining trials contained the same visual stimulus and a two-photon photostimulus (duration of 500 ms, triggered 500 ms after visual stimulus onset) of a single target cluster (V + P trials). Each cluster was stimulated in 52 to 175 (mean = 133.4 ± 25) trials.

### Ultrasparse dendritic imaging

Ultrasparse expression of calcium indicator within a Cre-recombinase-expressing population of pyramidal neurons was achieved using the virus mixture: AAV2/1-*Ef1a*-DIO-FLPo (gift from L. Zhang, Addgene viral prep, 87306-AAV1) diluted 1:75,000 to 1:100,000 from a stock concentration of 1.4 × 10^13^ g.c. per ml and AAVDJ-Ef1a-fDIO-GCaMP6s (gift from K. Deisseroth, Stanford, AAV-165) diluted 1:9 from a stock concentration of 7.0 × 10^12^ g.c. per ml. Dilutions reported are final. A total of 100 nl of virus was injected into each of two to four locations positioned about 500 µm apart in V1 of *Tlx3-cre* mice. High-magnification apical tuft imaging (Fig. 3) was performed on a commercial two-photon microscope (Neurolabware) using the Coherent Chameleon Discovery laser and the Nikon 16×/0.8 NA objective. In some experiments, an ETL (Optotune) was used to extend the length of dendritic branch simultaneously imaged. Imaging was always performed at 13.2 Hz final framerate using 920 nm excitation. The experimental flow is illustrated in Extended Data Fig. 4. Lower-magnification-volume imaging of apical and basal dendrites (Extended Data Fig. 8) was performed using the 10×/0.5 NA objective (Thorlabs) mounted onto a resonant scanning microscope (Ultima 2P plus, Bruker). An ETL (Optotune) was used to image four planes at 24 Hz total frame rate using 920 nm excitation.

### Two-photon optogenetic stimulation and dendritic imaging

We developed a new strategy for simultaneous ultrasparse two-photon dendritic imaging in V1 and two-photon optogenetic stimulation in LM. Expression of calcium indicator and opsin were achieved by injecting the same virus mixtures and dilutions as above for two-photon optogenetic stimulation during population imaging, and dendritic imaging of apical tufts with ultrasparse expression, with one difference. Expressing calcium indicator in LM neurons causes their axons to fluoresce in layer 1 of V1, interfering with sparse imaging of the apical dendrites of V1 neurons. To avoid this problem, the calcium indicator virus injections were targeted to V1 only, using blood vessel patterns as a guide. The opsin virus injections were targeted to LM only to avoid unintended activation of V1 neurons. The expression locations were later confirmed using retinotopic mapping. Experiments were performed using the same equipment as the two-photon optogenetic stimulation and population imaging experiments above, with a 10×/0.5 NA objective. An image of the brain surface over the two-photon FOV was affine transformed onto the brain surface image obtained previously in wide-field intrinsic imaging. The wide-field retinotopic map was then used to assign a retinotopic location for each position in the two-photon FOV. For the experiments that produced the non-specific suppression results shown in Fig. 4a–d, for every dendrite recorded, 9 groups consisting of 25 targets each were stimulated for 15 to 25 trials (mean, 23) with a photostimulation duration of 250 ms. In feedback-recipient spine detection experiments that produced at least one spine, between 308 and 2,994 targets (mean, 2,146) were assigned as a grid in LM, overlapping the retinotopic location of the imaged neuron in V1, also determined from the wide-field map. Targets spanned 2 *z*-depths, when C1V1 expression was restricted to layer 5 using *Tlx3-cre* (6 spines, 5 recordings, 2 animals), or 4–6 *z*-depths when C1V1 expression was Cre-independent and spanned the depth of LM (28 spines, 21 recordings, 9 animals). Targets were stimulated in random groups of 8 to 25 targets (mean, 22), with each group stimulated only once. A total of 420 to 1,260 unique groups (mean, 1,066) were used per experiment, in which every target participated in 8 to 16 groups (mean, 12). Stimulation was performed with 12–16 mW per target every 1.25 s (21 recordings) or 2 s (5 recordings) and lasted 500 ms. The imaging and stimulation scan paths were configured to be parfocal, with the SLM addressing light to stimulate spots both above and below its focal plane. The imaging plane was moved along the *z*-axis to image either somatic or dendritic signals using an ETL. Dendritic imaging FOVs spanned 80 to 150 µm to a side. Responses were recorded for 15 to 25 min using 30 to 50 mW average power, and imaging data were rigidly registered as they were acquired to correct for motion artifacts online.

### Procedures for online detection of feedback-responsive spines

Feedback inputs in the visual cortex are known to make contacts with both apical and basal dendrites of L5 pyramidal neurons^4,65,66^. We therefore searched for feedback-responsive spines in apical dendrites using the following strategy. The registered recordings and their average images were used to place small elliptical ROIs over all protrusions from the dendrite that could represent a spine. ROIs were assigned and fluorescence-extracted using ImageJ. These traces were transformed to (*F* − *F*_0_)/*F*_0_ where *F*_0_ was defined as the 10th percentile of a 90 s moving window. A stimulus response for each ROI on each trial (∆*R*) was calculated by averaging fluorescence in the nine frames before each stimulation and subtracting it from the average fluorescence in the seven frames after the offset of each stimulation, avoiding any stimulation artifact. Independent spine activity was used to identify spines that were potentially driven by stimulation. ∆*R* for each spine across trials was compared to the average ∆*R* across all spines (∆*R*_mean_). As most activity was correlated across spines, these plots often contained diagonally extended distributions of data (Extended Data Fig. 9d). Independent spine activity was visible as a cloud of points with high ∆*R*, and ∆*R*_mean_ around zero. For each spine, the group of targets stimulated on trials that passed a threshold set on ∆*R* but stayed below a threshold on ∆*R*_mean_ was identified. If any targets were stimulated on more than 20% (reliability threshold) of these trials, they were further collected for inspection of all of the trials on which those targets were stimulated, regardless of whether they generated independent activity. This analysis was then repeated iteratively, while varying the thresholds set on ∆*R* (mean + 0.5 s.d. to 3 s.d.), ∆*R*_mean_ (mean + 0.5 s.d. to 1.5 s.d.) and the percentage reliability (10 to 20%). This process reduced the number of possibly effective target–spine combinations to a number that could be visually inspected online. Among these possible connections, the 1 to 15 (mean, 8.3) most promising candidates were selected on the basis of the overall reliability and temporal profiles of the responses. New stimulation groups composed of these targets were designed to confirm whether any were indeed connected. In early experiments (*n* = 2) these confirmation blocks were composed of 20 min of recording in which 22 random combinations of the selected targets were stimulated, and the experiment ended there. Data from these confirmation blocks were used for the boosting analysis in Fig. 4 in these cases. It was found that most selected targets were not effective in driving spine activity but a small number were. Thus, in later experiments, spine signals from shorter, five to ten minute confirmation blocks were analysed online, and spines that were reliably responsive to stimulation were identified. New target groups were then designed to stimulate only those confirmed target–spine combinations, either with or without additional visual stimulation. These data were then used for the boosting analysis. In a subset of experiments (*n* = 16) the experiment was started by mapping receptive fields using sparse noise stimulation and somatic imaging. When both a confirmed target–spine combination as well as a receptive field were obtained in the same neuron, feedback stimulation was combined with presentation of an inverse visual stimulus (Fig. 3) centred on that neuron’s RF. In another subset of experiments (*n* = 4), we also delivered sparse noise stimuli during feedback stimulation. These data are not shown separately.

### Semisparse dendritic imaging

Semisparse expression of calcium indicator was achieved (Extended Data Figs. 6, 7 and 11) using the following virus mixture: AAV2/1-Synapsin1-FLEX-GCaMP7s (gift from D. Kim and the GENIE Project, Addgene viral prep, 104491-AAV1) diluted 1:7 from a stock concentration of 1.5 × 10^13^ g.c. per ml and AAV2/1- or AAV2/9-CAG-FLEX-tdTomato (gift from E. Boyden, Addgene viral prep, 28306-AAV9) diluted 1:150 from a stock concentration of 2.1 × 10^13^ g.c. per ml. A total of 20–50 nl of virus was injected into each of four locations around 500 µm apart in V1 of *Tlx3-cre* mice. Imaging was performed using the same set-up as for apical tuft imaging (above). An ETL was used for volume imaging, with two planes positioned in layer 5 to capture somata, and two imaging planes around the bifurcation of apical dendrites at the layer 1 to layer 2/3 transition^67^. Imaging was performed at 6.6 Hz final framerate per plane using 920 nm excitation.

### Dual-colour two-photon imaging

Simultaneous expression of red calcium indicator^68^ and green GABA indicator^69^ was achieved using the following virus mixture: AAV2/1-Synapsin1-FLEX-NES-jRGECO1a (Addgene, 100853) diluted 1:2 from a stock concentration of 2.7 × 10^13^ g.c. per ml, and AAV2/1-CAG-FLEX-iGABASnFR.F102G (gift from L. Looger, Addgene, 112167, viral prep by Charité–Universitätsmedizin Berlin Viral Core Facility VCA-148b) diluted 1:2 from a stock concentration of 5.1 × 10^12^ g.c. per ml. A total of 500 nl of virus was injected into each of two locations in V1 positioned 1 mm apart. Simultaneous expression of red calcium indicator and green glutamate indicator^70^ was achieved using the following virus mixture: AAV2/1-Synapsin1-FLEX-NES-jRGECO1a (gift from D. Kim and the GENIE Project, Addgene viral prep, 100853-AAV1) diluted 3:4 from a stock concentration of 2.7 × 10^13^ g.c. per ml mixed with AAV2/1-CAG-FLEX-SF-iGluSnFR-A184S diluted 1:4 from a stock concentration of 1–5 × 10^12^ g.c. per ml (gift from J. Marvin and L. Looger). A total of 200 nl of virus was injected into each of four locations positioned about 300 µm apart in V1 of *Tlx3-cre* mice. Imaging was performed using the same equipment as for dendritic imaging, with the addition of a Coherent Fidelity-2 fibre laser for excitation of jRGECO1a at 1,070 nm. The lasers were co-aligned through one scan path and the total power was kept below 100 mW. FOVs were 250 µm to 400 µm wide. Volume imaging was performed using either a piezoelectric objective positioner (Physik Instrumente) or an ETL (Optotune). When using a piezoelectric objective positioner, one plane was acquired in layer 5 and one plane was acquired in layer 1. Layer 1 imaging planes were positioned 30 to 100 µm below the pia. When using an ETL, two planes were acquired in each layer. Imaging was performed at 6.6 Hz final framerate per plane.

### Visual stimuli

#### Receptive field mapping

Visual stimuli were generated using Psychophysics Toolbox and synchronized to imaging data post hoc using MATLAB or LabVIEW. After recovery, every animal underwent one preparatory wide-field imaging session in which retinotopic mapping was performed with drifting or flashing bars^71^. Wide-field imaging was performed using calcium fluorescence for all animals except for those used in the two-photon optogenetic stimulation during dendritic imaging experiments. These animals had calcium indicator expression restricted to V1 only. We therefore used intrinsic signals obtained under anaesthesia to produce retinotopic maps. This allowed localization of LM and V1 to guide the appropriate placement of two-photon imaging FOVs. Ultrasparse apical tuft imaging experiments were started with wide-field-map-guided FOV placement and proceeded with receptive field mapping using forward correlation. The stimulus used here was an 8 by 8 grid of 8° squares that transitioned from grey to black to white and back at 2 Hz for 2 s, one at a time on a grey background. Squares were visited in pseudorandom order, with a 1 s intertrial interval for a total of 8 times per square in one 10 min run. Receptive fields were calculated online by averaging deconvolved responses to each grid position, using data from one to three runs. Ultrasparse volume imaging experiments (Extended Data Fig. 8) were started with receptive field mapping using reverse correlation of responses to 40 min of 5% sparse noise stimuli comprising 6–7° squares in a 6 by 6 grid updated at 4 Hz. In optogenetic stimulation during population imaging, semisparse dendritic imaging and dual-colour imaging experiments, two preparatory imaging sessions were performed: one wide-field imaging session and one two-photon imaging session to produce retinotopic maps at cellular resolution. In these cases, retinotopic mapping was performed with a 5% sparse noise stimulus composed of 6–7° squares in a 10 by 10 grid, where randomly chosen squares transitioned at 4 Hz from grey to white or grey to black. Between 30 and 60 min of data were obtained. For optogenetic stimulation during population imaging, these data were used to build cellular-resolution maps to guide stimulation group positioning and analysis. For the remaining experiments, this preparatory session allowed approximate positioning of visual stimuli for each FOV. Each experimental session of semisparse dendritic imaging and dual-colour imaging experiments was ended with 30 min of sparse noise stimulation to map receptive fields precisely. Receptive fields were calculated offline and neurons included or excluded from consideration based on how well the stimuli aligned with their receptive fields. For two-photon optogenetic stimulation during dendritic imaging, wide-field retinotopic maps were used to position stimulation targets and the recorded neurons in the visual field as LM neurons did not express indicator.

#### Drifting gratings

Visual stimuli were delivered with spherical correction applied^71^. In optogenetic stimulation experiments, visual responsivity was determined before assignment of stimulation groups using full-field sinusoidal gratings of 0.02 or 0.08 cycles per degree (c.p.d.) spatial frequency drifting in one of eight directions (0°, 45°, 90°, 135°, 180°, 225°, 270°, 315°) at a 2 Hz temporal frequency. During stimulation, a full-field grating was displayed (0.05 c.p.d., 2 Hz) drifting in one of four directions—0°, 90°, 180°, 270°—for 2 s, followed by a 4 s baseline period. In ultrasparse imaging experiments (Fig. 3 and Extended Data Fig. 8), five different shapes of sinusoidal grating (0.02–0.08 c.p.d., 2 Hz, 8 directions) were used as stimuli: an 8° Gabor patch, a 16° Gabor patch, an inverse stimulus consisting of a 16° inverse Gaussian transparency mask on a full-field sinusoidal grating background, an ‘annulus’, consisting of an 16° inner inverse Gaussian mask and a 28° outer Gaussian mask and, finally, a full-field stimulus. Stimuli were on for 1 s, followed by a 2.5 s baseline period. In semisparse dendritic imaging experiments (Extended Data Figs. 6, 7 and 11), Gabor patches (2 Hz, 0.05 c.p.d., 4 directions) of six sizes (5°, 10°, 20°, 40°, 60° and full field) were used. For dual-colour GABA-SnFR experiments either a 20° Gabor or full-field gratings (2 Hz and 0.05 c.p.d., 8 directions) were used. For dual-colour Glu-SnFR experiments, only full-field gratings (2 Hz, 0.08 c.p.d., 8 directions) were used. In semisparse dendritic imaging and dual-colour imaging experiments, stimuli were displayed for 1 s and followed by a 1.5 s baseline period. In dual-colour imaging experiments, the red channel of the monitor used for stimulation was turned off to avoid imaging artifacts. Stimuli were delivered using two separate monitors: ACER B276HL (1,920 × 1,080 px, 60 Hz) for two-photon stimulation and ultrasparse volume imaging, and ASUS VG278HV (1920 × 1080 px, 144 Hz) for all of the other experiments. Monitors were positioned 20 cm away from the mouse, at approximately 30° to the mouse’s midline in the right hemifield. All sinusoidal gratings were 62% contrast.

### Calcium imaging data preprocessing

Two-photon calcium imaging data were motion-corrected, segmented and fluorescence-deconvolved where indicated using Suite2p^72^ in all experiments except for all-optical spine mapping, in which ImageJ was used for online ROI selection. Deconvolution time constants were measured from the data. Data are presented as mean ± s.e.m. unless otherwise indicated.

### Receptive field calculation

For offline (post-experiment) sparse noise receptive field mapping, neuropil-subtracted and deconvolved event traces were used. Event traces were denoised by thresholding at twice their s.d. over their mean. Event-triggered stimulus ensembles were generated for every neuron by collecting the stimuli that preceded each event in a 2 s period and weighting those stimuli by the size of each event they preceded. The mean over event-triggered stimulus ensembles was calculated for that 2 s window and the value in each stimulus frame and sparse noise grid position (10 × 10) was expressed as a *Z*-score over all stimulus frame and grid position combinations and median filtered within frame. A neuron’s retinotopic preference was determined by the location of the maximum *Z*-score in a 600 ms window positioned over the peak of the event triggered average over time. This analysis was performed separately for light increments and decrements to get ON and OFF receptive fields. ROIs that did not produce a maximum *Z*-score of five in either ON or OFF maps were excluded when making maps for two-photon stimulation experiments, and a maximum *Z*-score of two when including neurons for analysis in semi-sparse dendritic imaging. These thresholds were chosen by inspection and varied because variability in the data differed based on the magnification of imaging. If both stimulus types were above the threshold, the retinotopic preference was computed as a weighted average of the two. For semisparse dendritic imaging experiments, neurons were further included for analysis based on the proximity of their receptive fields to the centre of sinusoidal gratings displayed. The results were robust to changing this distance criterion, which is noted in the figure legends. For dual-colour imaging experiments, receptive fields were calculated for the population to confirm that stimulation covered receptive fields, but no exclusion criteria were applied. When map-making for two-photon photostimulation experiments, all neurons passing the inclusion criteria were plotted in 3D such that their azimuth or elevation positions were the third dimension and their position in the FOV was the first two. This cellular resolution retinotopic preference map was used to fit a smooth surface model that was used as a template for receptive field approximation in subsequent photostimulation experiments. Fitting of elevation and azimuth maps was performed semi-automatically using a LOWESS surface fit (polynomial: linear, span: ~20, robust: bisquare) using the cftool in MATLAB. The surface fits were used to infer receptive field positions for optogenetic experiment targeting and analysis after ROI coordinates were corrected for two effects. First, they were transformed to compensate for magnification changes associated with ETL engagement. Second, ROI coordinates were transformed to compensate for FOV changes across imaging sessions using affine transformations fit to match surface blood vessel patterns recorded in receptive field mapping sessions and photostimulation sessions. Finally, we used the centroid locations of responding neurons to infer their azimuth and elevation preferences from the fitted surface models. For online receptive field mapping (both forward and reverse (Fig. 3 and Extended Data Fig. 8)) fluorescence was extracted by hand-drawn ROIs and then deconvolved. Receptive field position was decided visually, either at the location of the grid position eliciting the strongest response, or at the midpoint between the ON and OFF positions eliciting the strongest responses.

### Detection and mapping of two-photon stimulation responsive neurons

Segmentation results were manually inspected. Fluorescence traces from segmented ROIs were converted into a (*F* − *F*_0_)/*F*_0_ representation, where *F*_0_ was assigned as the 10th percentile of all samples in a 2,000 frame rolling window. The response of every neuron on every trial was represented as a signal-to-background ratio. First, the mean (*F* − *F*_0_)/*F*_0_ value in a 500 ms window after the end of photostimulation (or, for visual-stimulus-only trials, the point at which photostimulation would have ended had there been any), was computed (*S*_*i*_, for trial *i*). Next, the mean (*F* − *F*_0_)/*F*_0_ value in a 500 ms window preceding visual stimulus onset was computed (*B*_*i*_). Finally, the difference *S*_*i*_ − *B*_*i*_ was divided by the s.d. of the *B*_*i*_ values over trials i-2, i-1 and i. We call this value *R*_*i*_^V+P^ if during that trial both a visual stimulus and a photostimulus were delivered, and *R*_*i*_^V^ if only a visual stimulus was delivered. For every stimulated cluster separately, significantly responsive neurons (responders) were detected in two steps. First, after randomly sampling of *R*_*i*_^V^ trials to match the proportions of visual stimulus orientations between *R*_*i*_^V+P^ and *R*_*i*_^V^ trials, a Wilcoxon rank-sum test was performed between the two trial types (*R*_*i*_^V+P^ and *R*_*i*_^V^). Running different random samples did not change the ultimate result. Second, multiple comparisons were corrected for by controlling the FDR using mafdr in MATLAB^73,74^. The results reported in Figs. 1 and 2 are for an FDR of 2.5%. Our results do not depend on this threshold qualitatively (Extended Data Fig. 2). Next, the retinotopic preference of each neuron was estimated by interpolation from a smoothed retinotopic map generated in a previous imaging session (Figs. 1c and 2a) and aligned to the current session by affine registration of the brain surface blood vessel pattern. Any ROIs within 75 µm to either side of the V1/LM border estimated from this map were excluded from consideration. Next, to reveal the retinotopic distribution of detected responders, their retinotopic distance from the photostimulated location was calculated as every responder’s pair-wise retinotopic distance to all facilitated responders in the photostimulation area (source neurons; Extended Data Fig. 2). All locally facilitated responders were considered to represent the stimulated retinotopic location, as both photostimulus-driven and synaptically driven neurons constitute potential input sources to the other area. The resulting retinotopic distances of local responders are highly correlated with the absolute physical distances due to the retinotopic organization of visual cortex, whereas across-area responders, even if retinotopically aligned, are a minimum of 150 µm and, in most cases, hundreds of µm away from the nearest source neuron. The resulting pairwise retinotopic distance probability distribution was binned into ~1.2° bins in visual space and normalized to a null distribution of the same kind, calculated by sampling all segmented neurons in the appropriate area 20,000 times (Extended Data Fig. 2). This normalization step was necessary because the availability of neurons at any given retinotopic distance from the locally facilitated responder population varied widely depending on expression density, the target locations, FOV size and positioning relative to the retinotopic map, and blood vessel distribution. This process was repeated separately for each target cluster (distributions for one example stimulation group are shown in Extended Data Fig. 2b). Finally, these weighted probabilities were smoothed with a moving average of 5 bins and averaged across all stimulation groups (Fig. 2b). The bias between the topographic distribution of facilitated and suppressed responders was assessed by first computing for each stimulation group and each sign of influence the centroids of the responder distributions obtained. A Wilcoxon rank-sum test was then performed between the centroid distributions of facilitated and suppressed responders across the stimulation groups (Fig. 2d and Extended Data Fig. 2e). The strength and retinotopic spread of locally facilitated responders overlapped but were not identical in V1 and LM. To exclude the possibility that this accounted for the finding of a retinotopic difference between facilitated and suppressed neurons in the feedback direction, a subsampling procedure was performed to equalize the stimulation strength of the two directions, and retinotopic differences were reassessed (Extended Data Fig. 3). We characterized all photostimulation groups by the number of local responders that they generated (stimulation strength), and binned them according to this metric (Extended Data Fig. 3a (left)). The distributions of responder numbers differed between the feedforward (stimulate in V1) and feedback (stimulate in LM) direction. We next sampled the overlapping part of the two distributions: for each bin that contained both V1 and LM stimulation groups, we randomly selected half the number of stimulation groups produced by the direction with fewer groups contributing to this bin (for example, for a bin that contained ten LM stimulation groups and six V1 stimulation groups, we randomly chose three LM and three V1 groups). The resulting resampled dataset contained the same number of V1 and LM stimulation groups, which also have the same (matched) distribution of stimulation strengths (Extended Data Fig. 3a (right)). From this matched dataset, we then recomputed the spatial organization of interareal influence (Extended Data Fig. 3b) and measured the difference between the centroids of across-border facilitated and suppressed neurons separately for V1 stimulation groups (feedforward) and LM stimulation groups (feedback). This procedure resulted in a single value per direction, describing the retinotopic displacement between facilitation and suppression in this subsample of stimulation groups. Finally, we repeated this matching procedure 5,000 times, selecting different random subsets of stimulation groups in the overlap of stimulation strength distributions, resulting in 5,000 measurements of retinotopic displacement per direction. Extended Data Fig. 3c shows the proportion of resampled datasets (out of the 5,000) that produced a negative retinotopic difference between facilitation and suppression (suppressed responders at higher distances than facilitated responders), separately for each direction. In the feedback direction, a negligible proportion of these resamples (*P* < 0.025) produced such a negative difference, while a substantial proportion did in the feedforward direction (*P* ≫ 0.025). This indicates that the suppressive-centre facilitating-surround profile of feedback was maintained in stimulation-strength-matched samples, and was therefore not caused by differences in local stimulation strength. We then repeated this analysis, equalizing retinotopic spread and physical distance to across-area responders, neither of which abolished the effect (Extended Data Fig. 3).

### Spatial extent and magnitude of photoactivation response in the targeted area

The spatial extent of the photostimulation was quantified using an approach similar to previously published methods^64^. Responses to photostimulation were represented as the probability of obtaining ‘a significant trial’ on a per neuron-target group combination (Extended Data Fig. 1a (top)), each *R*_*i*_^V+P^ was represented as a *Z*-score relative to all *R*^V^ on a neuron-by-neuron basis. The number of trials crossing *Z* = 1.64 (single tail *α* = 0.05) divided by the total number of trials yielded a response probability (*P*_response_). All distances were measured as the Euclidean distance in 3D. For axial measurements, only ROIs that had a target within one lateral HWHM (20.7 µm) were taken into account and the axial distance was measured as the *Z*-offset to this target.

### Effect of locomotion on population activity in V1 and LM

Styrofoam wheel motion was recorded using quadrature encoders (Kubler). Wheel displacement traces were differentiated and filtered with a 2.5 s moving average. Any trial of stimulus presentation in any experiment was assigned as a locomotion trial if the running speed during that trial exceeded a threshold of 3 cm s^−1^. Two datasets were combined for this analysis: visual stimulus only (V-type) trials from the functional connectivity experiments (*n* = 42 sessions) described above and V-type trials from a separate set of optogenetic connectivity experiments (*n* = 32 sessions), the same as the above in every respect other than that we presented 20° gratings during photostimulation instead of full-field gratings. For this analysis, the visual response of every recorded neuron was represented as the difference *S*_*i*_ − *B*_*i*_ as described above. Neurons were included for comparison based on retinotopic representation, visual response magnitude and reliability. Neurons were required to have retinotopic locations within 30° of the stimulus centre. For full field stimuli, the centre was defined as the centre of a very large Gaussian mask that was present over the stimulus but had negligible effect on the visible contrast. Neurons were required to pass a Wilcoxon signed-rank test for visual responsiveness (*B*_*i*_ versus *S*_*i*_) at *P* = 0.01 after Bonferroni correction, for at least one of the two stimuli displayed. Furthermore, neurons were required to have responded significantly on 30% of trials, with significance assessed using the threshold *Z* = 1.64 computed as above, under resolution measurement. To quantify the modulation of visually evoked responses with locomotion, a modulation index was computed per neuron as the difference between the mean response to the preferred stimulus of that neuron *R* = (*S*_i_ − *B*_i_) on locomotion trials and the mean response on stationary trials, normalized to the mean response on stationary trials (Δresponse = (*R*_run_ − *R*_sit_)/*R*_sit_). This value was averaged across all neurons in the same area within each session and a signed-rank test was performed between paired measurements of Δresponse from LM and V1 across all sessions.

### Ultrasparse dendritic imaging: local events in apical tufts

To identify local events^26,75–78^ in fine apical tuft dendrites of individual pyramidal cells expressing GCaMP using our ultrasparse expression strategy, imaging data were motion-corrected and downsampled fourfold in time to produce videos for inspection. Events were required to involve at least two spines active simultaneously, along with the dendritic branch between them, in the absence of simultaneous activity on the proximal end of the imaged dendrite. On the basis of these criteria, two experimenters (M.F. and D.H.), blinded to the visual stimulus timing and type, inspected all of the videos acquired independently, and then resolved discrepancies to arrive at consensus on the location and timing of dendritic events. ROIs were then hand drawn over the dendritic segments where events were identified, and over dendritic segments proximal to those, to compare integrated fluorescence and confirm the presence of an event in the distal ROI and the absence of the event in the proximal ROI. Automated detection of these events proved challenging for several reasons. First, local events could occur anywhere along the dendrite and could have varying spatial extents. Second, local events had varying signal-to-noise and varying rise and decay times in comparison to global events recorded in the same branch. Third, many events were bright and clearly involved a single spine and its parent branch, but a second spine could not be unambiguously identified. Some of these events must also be true multispine events in which the additional spines were not spatially resolved by our imaging, but we chose to be conservative and excluded any such events in which a second spine was not identifiable. Together, these features made the automated assignment of spatial ROIs, thresholds and other exclusion criteria a high dimensional task poorly constrained by the limited number of local events that we found (Extended Data Fig. 4). To measure the spatial extent of local events, two separate ROIs were drawn over each local event location. One ‘mask’ ROI encapsulated the entire branch with its spines, and the other ‘line’ ROI traced a single pixel-wide line along the branch only. The fluorescence measured in each pixel in the mask ROI was averaged into the nearest pixel of the line ROI. The geodesic distance between each line ROI pixel and the most proximal line ROI pixel was measured. The mean fluorescence over time in each resulting 1-px-wide geodesic distance bin was smoothed with a 4 s moving average and then converted to (*F* − *F*_0_)/*F*_0_, where *F*_0_ was assigned as the 10th percentile of the fluorescence in a moving window 45 s wide. This trace was then normalized to the s.d. of the whole trace over time. Fluorescence was also smoothed across space within individual time bins with a 2 µm moving average. Finally, the spatial profile of each event was calculated by averaging across identified frames, and events aligned and normalized to their respective fluorescence peaks across space. An idealized local event was generated by averaging across events, which was then fit with the sum of three Gaussians.

### Ultrasparse dendritic imaging: volume imaging of apical and basal dendrites

Imaging data were motion-corrected offline using the two-step procedure of Suite2P (v.0.9.2), and ROIs were hand drawn to avoid cross-talk with nearby processes. For the five neurons that showed a significant positive effect, we confirmed that ROIs that were segmented and neuropil subtracted using Suite2P produced the same result. Visual responses were quantified as the peak difference in a seven-frame (~1 s) window starting three frames after visual stimulus onset in comparison to baseline measured in a four-frame window ending one frame before visual stimulus onset. Responses were averaged across all apical dendritic ROIs (in the two superficial-most imaging planes) and all basal ROIs excluding the soma (in the deepest imaging plane). From this point onwards, only trials that produced a visual response in the average of basal ROI signals that was greater than 20% of peak response in the average of basal ROIs were included in the analysis to focus on global events, similar to the analysis for semisparse dendritic imaging. The ratios between the apical and basal responses were quantified trial by trial. Responses to the two Gabor stimuli were pooled to construct a low-surround stimulus class and responses to the inverse and full-field stimuli were pooled for a high-surround class. The difference between the average ratios for these two stimulus classes was computed. To assess the significance of the ratio difference, the procedure was repeated 3,000 times, with trials shuffled between the stimulus classes, replicating the number of trials obtained for each class. Neurons that produced a difference of larger than 95% of shuffles were regarded as significant. To measure correlations between ROIs, the apical to basal ratios were computed taking the response of each apical ROI and comparing it to the average of all basal ROIs. This resulted in a vector of ratios for each apical ROI. The correlation between these vectors was computed for the last panel of Extended Data Fig. 8.

### Relative modulation of apical trunks in semisparse dendritic imaging

After motion correction and segmentation, ROIs identified in layer 5 were included for analysis as ‘soma ROIs’ if they produced a significant receptive field (see above), and were clearly a section through a soma and not an apical dendrite of a deeper soma. All ROIs segmented from our second most superficial imaging plane, which was placed just below layer 1, were included for analysis as ‘dendrite ROIs’. Our most superficial imaging plane often extended into layer 1 where apical dendrites had already ramified and the density of fluorescent processes was high. These data were excluded to analyse only sections through apical dendritic trunks of pyramidal neurons, with the goal of coming as close as possible to imaging the nexus across a population with variable nexus locations. Connected somata and dendrites were identified by examining correlations between fluorescence traces as well as the deconvolved event traces for every possible pairing of soma and dendrite ROIs (Extended Data Fig. 7). Any pair that exceeded a fluorescence trace correlation of 0.45 and an event trace correlation of 0.25 was assigned as ‘connected’. These thresholds were found to provide a conservative decision criterion, as illustrated by the example in Extended Data Fig. 7. Somata and dendrites identified in this manner were traceable through structural *z*-stacks, but not always unambiguously so, as even a slight density of expression commonly leads to crossings of neuronal processes at distances smaller than the imaging resolution. Changing the correlation thresholds to 0.55 (*F*) and 0.35 (events) did not alter the results qualitatively. Fluorescence traces were converted to (*F* − *F*_0_)/*F*_0_ where *F*_0_ was assigned as the mean fluorescence of that ROI across the entire recording duration. To measure the effect of visual stimulus size on dendritic activity, responses to the more effective orientation (two directions) were used. The response of every ROI on every trial was quantified as the modulation from the baseline, where the baseline was defined as the mean fluorescence in the three frames preceding stimulus onset and the response was defined as the mean fluorescence in the ten frames after stimulus onset. Next, the value for every trial was normalized to the peak fluorescence recorded from that ROI in any trial. The trials were then sorted and binned by the somatic activity level separately for each stimulus size. Binning was performed with 5% bin width, going from −40% of peak to 100% of peak. All trials from all neurons in each bin were then averaged to obtain one population level value each for soma and dendrite for each stimulus size and bin. Next, a two-way ANOVA was performed on population data to determine the effect of somatic activity, stimulus size and their interaction on dendritic activity (Extended Data Fig. 6c). To examine whether individual neurons showed dendritic size tuning, somatic influence was removed on a cell-by-cell and trial-by-trial basis. For this analysis, only trials in which the soma was active to 20% or more of peak were included, as the effects were visible at higher activity levels at which dendrites were more strongly activated, and responses were quantified as the peak fluorescence on a given trial relative to baseline, with the same time windows used as above. To summarize and remove the relationship between somatic and dendritic fluorescence, a linear model was fit to data from a single neuron across all stimuli and the residuals obtained for each trial. A one-way ANOVA was then performed to examine whether stimulus size had a significant effect on the residuals. The preferred size of dendritic residuals was taken as the size that produced the largest residual fluorescence value. Working on deconvolved events with event-wise analyses instead of fluorescence with trial-wise analyses, and alternative procedures for matching somatic activity across stimulus sizes, produced qualitatively similar results. To measure the effect of locomotion on apical dendritic activity, a similar approach was used. First, data were binned and normalized within neuron to produce a normalized population average. These data were subjected to a two-way ANOVA to determine the effect of somatic activity, locomotion and their interaction on dendritic activity. Next, locomotion effects were measured on a cell-by-cell basis after removal of somatic influence. This was done by fitting a linear model to data from a single neuron across all stimuli and behavioural states, obtaining the residuals of this fit for each trial, and performing a two-way ANOVA on these data to determine whether locomotion, stimulus size or their interaction had a significant effect. Again, only trials in which the soma was active to 20% or more of peak were included. Next, the average residual within the stimulus and behavioural state category was computed for each neuron, and post hoc tests were performed to examine whether locomotion had a significant effect for each stimulus category separately. Stimuli were pooled as smaller than preferred, preferred or larger than preferred. The preferred size was computed on data obtained during stationary states. Here, using deconvolved events instead of fluorescence and alternative methods to remove somatic influence produced the same qualitative results.

### Boosting analysis for dendritic imaging during two-photon stimulation

A boosting index was calculated to examine whether feedback stimulation caused calcium signals in a dendritic branch segment that extends beyond the feedback-recipient spine itself. Two sets of two ROIs were drawn. One ROI of each set was placed on a reference branch of the same neuron, not carrying a stimulated spine. The other ROI of each set was placed distally onto the stimulated branch, but excluded the identified feedback-recipient spine itself. In cases in which only one extended stretch of dendrite was imaged, the reference ROI was placed proximally on the stimulated branch, as far as possible from the spine. The two sets differed in that the stimulated branch ROIs were drawn either relatively closer to or relatively farther from the stimulated spine. The distributions of the shortest straight-line distance between the centroid of the stimulated spine and the nearest pixel of the stimulated branch ROI are shown in Extended Data Fig. 10c. A boosting index was then calculated by taking the ratio of the post-stimulus fluorescence value in the stimulated branch ROI to that in the reference branch ROI. Boosting indices were calculated separately for trials in which stimulation was performed and blank trials in which no stimulation was performed and then compared across the trial types to examine whether there was an effect of stimulation.

### Dual-colour two-photon imaging

Layer 5 segmentations were manually curated to remove poorly sectioned neurons. Data were acquired in 10 or 20 min blocks of trials, and the first three trials excluded to remove a fast bleaching component. To represent population activity, the deconvolved event traces were averaged across segmented ROIs. To represent apical and basal SnFR fluorescence, the fluorescence recorded across the entire FOV was averaged and converted to (*F* − *F*_0_)/*F*_0_ where *F*_0_ was calculated as the 10th percentile of *F* across all timepoints separately for each acquisition block of 10 or 20 min. When two planes were acquired in each layer, the SnFR signals from the two planes within layer were averaged together. For the majority of iGluSnFR and all iGABASnFR sessions, receptive fields were mapped with sparse noise stimuli to confirm the retinotopic representation was well stimulated by our monitor. For the remaining iGluSnFR sessions, wide-field retinotopic maps were used. The linear models presented in Extended Data Figs. 11 and 12 were fit to unequal numbers of stationary and locomotion trials (stationary: 371 ± 143, locomotion: 364 ± 98 trials mean ± s.d.). Resampling to match trial numbers did not change the results qualitatively. We excluded sessions that contained less than 150 trials of locomotion. Models were highly significant, and accounted for a moderate amount of variance, with an *R*^2^ = 0.292 ± 0.17 (mean ± s.d.) across 12 sessions. To assess the significance of interactions between locomotion and glutamate signals, we performed a Wald’s model comparison test, in which we compared the full model with a restricted model excluding both interaction terms. In total, 4 out of 12 sessions produced significance (Extended Data Fig. 11k (solid points)) but most sessions had a trend.

### Reporting summary

Further information on research design is available in the Nature Portfolio Reporting Summary linked to this article.

## Online content

Any methods, additional references, Nature Portfolio reporting summaries, source data, extended data, supplementary information, acknowledgements, peer review information; details of author contributions and competing interests; and statements of data and code availability are available at 10.1038/s41586-023-06007-6.

## Supplementary information

Reporting Summary

Supplementary Video 1 Local event observed in ultrasparse dendritic imaging. The video shows a single dendritic branch from a layer 5 pyramidal neuron in V1 with the proximal end identified. The recording is from a visual stimulation experiment and is downsampled fourfold in time from an acquisition rate of 26 Hz. During the recording, a single spine activation is seen first, followed by a local event involving multiple spines and a segment of the branch.

Supplementary Video 2 Global event observed in ultrasparse dendritic imaging. The video shows the same branch as in Supplementary Video 1. In this recording, a global event occurs, where the entire branch is active.

Supplementary Video 3 All-optical spine mapping experiment. The video shows two distal apical tuft dendrites of one neuron in V1. A spine responsive to photostimulation of a target location in LM is indicated by an arrowhead. Photostimulation times are indicated by a lightning symbol at the top left. The recording shows ten individual trials followed by an averaged response across 25 trials. The recording was downsampled fourfold in time from an acquisition rate of 30 Hz. The dendrite is the same recording as shown in Fig. 4f,h.

Supplementary Video 4 Photostimulation-induced local dendritic event. The video shows multiple apical tuft dendrites of one layer 5 pyramidal neuron in V1 with two photostimulation-responsive spines on the same branch, identified by pink arrowheads. Photostimulation is indicated by a lightning symbol at the top left. The recording shows three individual trials, of which the first two trials led to spine activations and the third trial led to an extended branch specific activation. This is followed by a global event with all of the branches active.

## Data Availability

Datasets supporting the findings of this study are available from the corresponding authors on reasonable request.

## Code availability

Custom code for SLM calibration and near-automatic photoactivation response mapping (NAPARM) are available at GitHub (https://github.com/llerussell/SLMTransformMaker3D and https://github.com/llerussell/Naparm).
