# Supplementary material for: Cortico-cortical feedback engages active dendrites in visual cortex
Source: Nature. 2023 May 3;617(7962):769–76. doi: 10.1038/s41586-023-06007-6 (PMC10244179; doi:10.1038/s41586-023-06007-6)
Supplement: Supplementary file 1 — Reporting Summary [file 41586_2023_6007_MOESM1_ESM.pdf]

nature research

Corresponding author(s): Michael Hausser

Last updated by author(s): Jan 23, 2023

Reporting Summary

Nature Research wishes to improve the reproducibility of the work that we publish. This form provides structure for consistency and transparency in reporting. For further information on Nature Research policies, see our Editorial Policies and the Editorial Policy Checklist.

Statistics

For all statistical analyses, confirm that the following items are present in the figure legend, table legend, main text, or Methods section.

n/a	Confirmed	
☐	☒	The exact sample size (*n*) for each experimental group/condition, given as a discrete number and unit of measurement
☐	☒	A statement on whether measurements were taken from distinct samples or whether the same sample was measured repeatedly
☐	☒	The statistical test(s) used AND whether they are one- or two-sided<br>*Only common tests should be described solely by name; describe more complex techniques in the Methods section.*
☐	☒	A description of all covariates tested
☐	☒	A description of any assumptions or corrections, such as tests of normality and adjustment for multiple comparisons
☐	☒	A full description of the statistical parameters including central tendency (e.g. means) or other basic estimates (e.g. regression coefficient) AND variation (e.g. standard deviation) or associated estimates of uncertainty (e.g. confidence intervals)
☐	☒	For null hypothesis testing, the test statistic (e.g. *F*, *t*, *r*) with confidence intervals, effect sizes, degrees of freedom and *P* value noted<br>*Give P values as exact values whenever suitable.*
☒	☐	For Bayesian analysis, information on the choice of priors and Markov chain Monte Carlo settings
☒	☐	For hierarchical and complex designs, identification of the appropriate level for tests and full reporting of outcomes
☒	☐	Estimates of effect sizes (e.g. Cohen's *d*, Pearson's *r*), indicating how they were calculated

Our web collection on statistics for biologists contains articles on many of the points above.

Software and code

Policy information about availability of computer code

Data collection	Dendritic imaging and dual-color imaging data were collected using Scanbox (v4.0, Neurolabware) and PrairieView (v5.5, Bruker Technologies). Simultaneous two-photon optogenetics and calcium imaging data were collected using PrairieView (Bruker) Blink (v1.1.3.528, Meadowlark) and custom-written MATLAB (2015a-2019b, Mathworks) code for SLM calibration and photostimulation pattern production. Psychophysics toolbox was used for visual stimulation and LabVIEW (2017, National Instruments) or MATLAB were used for post-hoc synchronization.
Data analysis	Calcium imaging registration and segmentation was performed using Suite2P (Pachitariu et al 2017) except for all-optical spine mapping, where we used ImageJ (v1.52g, NIH) for online ROI selection. All further analysis was performed using custom written MATLAB (2015a-2019b, Mathworks) code. All statistical comparisons were performed using built-in Matlab functions except for repeated-measures ANOVAs, which were performed in SPSS (IBM.)

For manuscripts utilizing custom algorithms or software that are central to the research but not yet described in published literature, software must be made available to editors and reviewers. We strongly encourage code deposition in a community repository (e.g. GitHub). See the Nature Research guidelines for submitting code & software for further information.

Data

Policy information about availability of data

All manuscripts must include a data availability statement. This statement should provide the following information, where applicable:

- Accession codes, unique identifiers, or web links for publicly available datasets
- A list of figures that have associated raw data
- A description of any restrictions on data availability

Datasets supporting the findings of the study are available from the corresponding author on reasonable request.

April 2020

Field-specific reporting

Please select the one below that is the best fit for your research. If you are not sure, read the appropriate sections before making your selection.

☒ Life sciences ☐ Behavioural & social sciences ☐ Ecological, evolutionary & environmental sciences

For a reference copy of the document with all sections, see nature.com/documents/nr-reporting-summary-flat.pdf

Life sciences study design

All studies must disclose on these points even when the disclosure is negative.

Sample size	No power analysis or other statistical methods were used to pre-determine sample sizes but our sample sizes were (similar to those in previous publications. (For dendritic imaging see refs 25, 57,67. For two-photon stimulation see ref 18,55,56). For connectivity mapping (Fig 1 and 2), independent dendritic event detection (Fig 3), feedback stimulation during dendritic imaging (Fig 4), dendritic volume imaging (Ext.Data Fig. 8) and dual color imaging (Ext. Data Fig. 11-13), no additional data was collected after the reported statistical result was obtained. For apical trunk imaging in Ext. Data Fig 6-7,11, a preliminary analysis on population averaged data was performed on a smaller dataset which showed the reported effects qualitatively. The dataset was then doubled in size before statistical analysis was performed using measurement of dendritic residuals. All statistical tests were two sided unless indicated in the Methods.
Data exclusions	For apical trunk imaging (Ext. Data Fig 6-7,11), responses to sinusoidal gratings were collected prior to receptive field mapping, and neurons with receptive fields farther than 20 degrees from the grating center were excluded. For dual color imaging experiments to measure the effect of locomotion, experiments producing less than 150 running trials were excluded from comparison. In analysis of connectivity mapping experiments, photostimulated groups which did not produce any locally facilitated responders ('source neurons') were excluded from analysis; this was the case for a single target group. Stimulated groups for which the multiple comparisons correction using the FDR procedure produced a prior of 1 were also excluded; this was the case for 27 target groups. For dendritic volume imaging (Ext. Data Fig. 8) one cell was excluded because of unavoidable crosstalk with overlapping processes of other cells.
Replication	All figures involved experimental series using multiple mice and reported results held across mice. No attempt at replication was made outside of the reported results.
Randomization	Randomization of animals to different groups is not relevant to our study as all mice used in individual experimental series had the same genotype and rearing conditions. Visual stimulus delivery was pseudorandomized.
Blinding	Because independent dendritic event detection was performed manually, the experimenters were blinded to visual stimulus timing and type, which was pseudorandomized in time, during inspection of the data.

Reporting for specific materials, systems and methods

We require information from authors about some types of materials, experimental systems and methods used in many studies. Here, indicate whether each material, system or method listed is relevant to your study. If you are not sure if a list item applies to your research, read the appropriate section before selecting a response.

Materials & experimental systems

n/a	Involved in the study	
☒	☐	Antibodies
☒	☐	Eukaryotic cell lines
☒	☐	Palaeontology and archaeology
☐	☒	Animals and other organisms
☒	☐	Human research participants
☒	☐	Clinical data
☒	☐	Dual use research of concern

Methods

n/a	Involved in the study	
☒	☐	ChIP-seq
☒	☐	Flow cytometry
☒	☐	MRI-based neuroimaging

Animals and other organisms

Policy information about studies involving animals; ARRIVE guidelines recommended for reporting animal research

Laboratory animals	Male and female adult mice (P50-P120) were used in experiments and kept on a C57BL/6 background and were of the following genotype: Tlx3-Cre PL56 (GENSAT) or Tlx3-Cre PL56; CaMKII-tTA (Jax #007004) ; TITL-GCaMP6s (Jax #024104). Animals were kept at a normal 12hr light/dark cycle at a temperature of 22oC and 62% humidity.
Wild animals	No wild animals were used in this study.
Field-collected samples	No samples were collected in the field.

Ethics oversight	All experimental procedures were carried out under license from the UK Home Office in accordance with the UK Animals (Scientific Procedures) Act (1986).

Note that full information on the approval of the study protocol must also be provided in the manuscript.
